# A novel fuzzy framework for technology selection of sustainable wastewater treatment plants based on TODIM methodology in developing urban areas

**DOI:** 10.1038/s41598-022-12643-1

**Published:** 2022-05-25

**Authors:** Gunes Eseoglu, Kozet Yapsakli, Hakan Tozan, Ozalp Vayvay

**Affiliations:** 1 Engineering Management Department, Graduate Institute for Pure and Applied Sciences, Marmara University, Goztepe, Istanbul, Turkey; 2 Environmental Engineering Department, Faculty of Engineering, Marmara University, Goztepe, Istanbul, Turkey; 3 Department of Industrial Engineering, Faculty of Engineering and Natural Sciences, Istanbul Medipol University, Kavacik, Istanbul, Turkey; 4 Department of Industrial Engineering, Faculty of Engineering and Natural Sciences, Istanbul Health and Technology University, Merter, Istanbul, Turkey

**Keywords:** Environmental sciences, Engineering

## Abstract

Optimal technology selection of wastewater treatment plants (WWTPs) necessitates the adoption of data-driven scientific approaches that satisfy the sustainability requirements of the urban ecosystem. Such approaches should be able to provide actionable insights to decision makers constrained by factors such as population growth, land scarcity, and loss of functionality of wastewater treatment plants. The framework in this study proposes a hybrid fuzzy multi-criteria decision making (MCDM) model consisting of the analytical hierarchy process (AHP) and the TODIM (an acronym in Portuguese of interactive and multi-criteria decision-making) by using alpha cut series which takes into account the risk aversion of decision makers (DMs) to overcome uncertainties of environmental conditions. The literature to date indicates that the study is the first to presents how a systematic decision-making process is approached by interpreting the interaction of criteria for the selection of wastewater treatment technology through the membership function of Prospect Theory. The proposed methodology reveals that the prominent reference criterion manipulates other sub-criteria according to the function of risk-aversion behavior. The fuzzy sets based on alpha cut series are employed to evaluate both the criteria weight and the rank of the alternatives in the decision-making process to obtain compromise solutions under uncertainty. The dominance degrees of the alternatives are achieved by fuzzy TODIM integrated with the fuzzy analytic hierarchy process (FAHP) which deals with the uncertainty of human judgements. According to the ranking results determined by the dominance degree of alternatives, anaerobic–anoxic–oxic (A2O) without pre-clarification was the most effective process in relation to the sludge disposal cost (C25) calculated as reference criteria. The ranking of four full-scale WWTPs in a metropolitan city of an EMEA country based on 24 sub-criteria listed under the four main criteria, namely the dimensions of sustainability, is used as a case study to verify the usefulness of the fuzzy approach. Motivated by the literature gap related to the failure to consider the psychological behavior of DMs in technology selection problem for wastewater treatment, it is discussed how the proposed hybrid MCDM model can be utilized by reflecting human risk perception in wastewater treatment technology selection for developing urban areas.

## Introduction

Economic development should be balanced with protection of natural resources and environmental sustainability to contribute to the circular economy. Wastewater is defined as a valuable resource for both ecological and economic development of countries in terms of sustainability^1^. The increasing importance of sustainable urbanization in developing countries and the rapid depletion of resources with the increasing population require a rational assessment of the need for wastewater treatment plants and the sustainability of existing facilities in order to minimize the risks arising from the possible water crisis in the near future. Wastewater treatment makes a major contribution to sustainable development in terms of water resources protection, effective waste management, and openness to the use of renewable energy^2^. Increasing the proportion of safe and most appropriate technologies for treating domestic and industrial wastewater is considered one of the expanded goals of the Sustainable Development Goals (SDG) by 2030^3^.

Optimal technology selection for the wastewater treatment process can only be achieved by ensuring the right investment for the right region, taking into account public benefit and social awareness. In real life, the optimal technology selection for wastewater treatment plants depends directly on the knowledge, experience and competence of the decision makers^4^. Uncertainties related to forecasts of economic, social, and environmental conditions can have manipulative effects on stakeholders' attitudes (e.g., risk aversion or risk-taking in making decisions) and lead to limitations in rational decision-making that requires sufficient knowledge of prevailing conditions^5^. For this reason, the motivation of this study is to overcome the limitations of the unpredictability of human behavior caused by uncertainty by reflecting the risk-aversion perspectives of competent decision-makers in a proposed decision-making model for selecting the most appropriate technology for wastewater treatment plants (WWTPs). In general, real world MCDM problems related to environment should be viewed as fuzzy problems in nature, including objectives, dimensions, attributes, and alternatives^6^. Decision-makers compare the two alternatives based on qualitative and quantitative data and decide on the suitability of a wastewater treatment technology in relation to different professional experiences such as environmental impact assessment, construction, design and, operation. When evaluating perspectives from different experiences, linguistic data is used that reflects the qualitative, since the dominance of one technology over another cannot be expressed in crisp values^7^. Furthermore, expressing the criteria weights, the evaluators’ weight, and the evaluators’ judgement as linguistic variables is the more preferred way than quantitative data to highlight the complexity of socio-economic and socio-cultural conditions, the applicability of technology, and the availability of innovations^8^. In order to cope with the uncertainty that results from the subjectivity of linguistic evaluations, linguistic data are expressed with fuzzy sets and made usable for mathematical operations^9^. Fuzzy sets enable not only the evaluation of alternatives, but also the expression of decision making criteria for weighting under uncertain environments^10^. Consistent with all this information, fuzzy set theory offers advantages in tolerating the ambiguity of human judgements, uncertainties and imprecise or insufficient information regarding quantitative and qualitative data.

Wastewater treatment technology selection is a complex and multidimensional problem necessitating multi criteria evaluation^11^. In addition to the complexity of the problem, decision makers are faced with the necessity of evaluating contradictory criteria which, seems to be another challenge in the problem^12^. Accordingly, much research has been done to assess this interaction between economic or technological feasibility and environmental impacts in order to select the optimal wastewater treatment alternative over the lifetime of treatment systems. Among them, Molinos-Senante et al.^13,14^ propose a systematic approach based on the analytic hierarchy process AHP and scenario-based analytic network theory (ANP) to address the assessment of economic feasibility and environmental impact in the decision-making process for ranking alternatives. In recent years, most studies on the economic assessment and environmental impacts of alternatives to wastewater management, including wastewater treatment plants, have been conducted by applying a multi-criteria decision-making approach (MCDM) that focused on expert opinion^15^. Furthermore, some of these studies should be conducted with exact data as opposed to heterogeneous data, however, this is not always possible with real-world problems. All these mentioned studies contribute with different perspectives to deal with the complexity of the decision-making process for the technology selection of wastewater treatment plants and uncertainties from dynamic environments.

In this article, the authors focus on the impact of behavioral psychology on the decision-making process, considering the possibility of reference dependency, loss-aversion, and subjective judgement bias to select the optimum technology for WWTPs under risks and uncertain environments. One of the reference studies in behavioral economics is the Prospect Theory developed by Kahneman and Tversky in 1979^16^, a descriptive model that includes behavioral expectations for individual decisions under risk conditions. Prospect theory treats changing individual behavior under risk and uncertainty as a description of reference-dependent losses and gains. Although the effect of behavioral psychology on decision-making processes is repeatedly emphasized in the literature on wastewater management, it describes it qualitatively in decision-making models. On the other hand, it is possible to use this mechanism in quantitative decision models via prospect theory to make more rational decisions. In this context, Autran Monteiro Gomes and Duncan Rangel in 2009^17^ introduced an MCDM method based on prospect theory and considering psychological behaviors under risk and uncertainty, known as the TODIM method (an acronym in Portuguese for interactive and multi-criteria decision-making) is abbreviated. Q. Qin et al. in 2017^18^ introduced a TODIM-based approach to behavioral decision making integrated with an intuitionistic fuzzy set to transform linguistic data for business model selection related to energy efficiency. Guo et al. in 2020^19^ improved an extended TODIM methodology using a hesitant fuzzy set to select carbon capture, utilization and storage technologies to represent ambiguity in decision making.

Starting from this perspective, a fuzzy multi-criteria decision framework based on Prospect Theory is introduced to integrate bounded rational human behavior emphasized by many studies in behavioral economics on the decision model for WWTP selection problem. Fuzzy logic- based methodology deals with heterogeneous data (quantitative and qualitative) under uncertain environment and ambiguity from subjective judgements. The decision-making framework proposed in this study, including fuzzy AHP used to assess subjective judgements of evaluators, and the fuzzy logic-based TODIM methodology ranked the alternatives by reflecting the risk behavior of decision makers. The α-cut series used to capture compromise solution holds fuzzy information throughout the decision-making process. The normalized phase of the subjective and objective data converted into fuzzy information is carried out using the derived method developed by Abdel-Kader^20^. In order to make more rational decisions and handle uncertain environment hybrid methods are more useful and reliable tools due to well-organized and integrated solution mechanisms for required tasks such as weight evaluation, aggregated weighting, ranking of alternatives and achievement of compromise solutions in MCDM process^12,21–23^. The other advantages of hybrid models in multi-criteria decision problems are: Ability to use the integration of the capabilities of more than one technique to solve complex problems involving variety of information and, to transform both quantitative and qualitative data into aggregate weights to place them in a membership function^24–27^.

In MCDM problems, weight determination is the key phase, divided into weighing subjective judgements and objective data. As a strategic part of the proposed decision-making framework, the subjective judgments of an evaluation consortium composed of four different perspectives with four different professional experiences are converted into trapezoidal fuzzy numbers (TrFNs), and fuzzy AHP is applied to determine criteria weights. First, AHP presents a clear hierarchical viewpoint to compare priority of performance indicators in decision making problems related to the relationship or interaction between criteria. AHP combined with fuzzy logic manipulates the imprecise judgements, ambiguity of human thinking and, uncertain environment during assessment of criteria weight and, aggregate the expert scores for criteria into fuzzy sets. Fuzzy AHP demonstrates the relative importance for pairwise comparison, measures the consistency ratio to ensure valid pairwise judgements and, allows defuzzification of fuzzy weights to obtain the final criteria score^28^. Furthermore, fuzzy AHP can be easily applied as one of the techniques in hybrid decision models to complex decision making problems involving large criteria and subjective judgments, since it performs in agreement with other MCDM methods.

In contrast to traditional decision making tools, the methodology proposed in this study is worth showing the results of reference-dependent decisions that reflect loss-averse behavior and ranking the alternatives according to their degree of dominance by showing the criteria interaction. Additionally, the criteria system is established including all sustainability dimensions that allow comprehensively analyze not only single effect of specific environmental impact such as greenhouse gas effect or treated water reuse but rather the whole operations from the construction to operation. The diverging aspect of this study from the other studies on wastewater management is that in the decision making process, sustainability indicators determine the dominance degree of the technologies used in real-scale WWTPs in accordance with the membership function of the Prospect Theory. The strongest aspect of this framework is that it enables more rational decisions by determining the crucial criteria that influence the concept of sustainability under variable and uncertain conditions according to the risk aversion approach. Based on this perspective, the results the authors have obtained according to the proposed decision-making model which takes into account the behavioral characteristics of DMs, reveal the strong interaction between economic and environmental criteria in real scale decision-making problems for WWTPs, and also provide a reduction in subjective bias judgements or loss of information. In this study, the reference dependency approach reflects the decision model based on the loss avoidance behavior of decision makers and the proposed decision-making model calculates the sludge disposal cost, which is a sub-criterion of the economic indicators as ‘’the reference criterion’’. The calculated reference criterion manages the decision-making model, interacts strongly with sludge generation, operation and maintenance cost, and energy saving criteria to determine the degree of dominance of each alternative. According to the dominance degree of alternatives, A2O (anaerobic–anoxic–oxic) without pre-clarification was the most effective process from the point of view of sustainability. All of these results suggests that while the model simulates the reference dependency of human behavior with a focus on risk aversion, the weight of the criteria that influence the weight of reference criterion is also effective in alternative ranking.

When the examining the contributions and limitations of researches on the environmental policy-making, the following conclusions seems to emerge:

1. The need for a multidimensional assessment of the problem by defining a criteria system,
2. Achievement of the evaluation of heterogeneous information types such as linguistic, interval or crisp data and demonstration of criteria interaction,
3. Dealing with uncertainties arising from dynamic environmental conditions and human prejudices,
4. Reflecting the behavioral psychology of DMs in the decision-making process quantitatively,
5. Management of the decision-making process by overcoming incomplete, corrupted or insufficient data.

Motivated by these inferences, a fuzzy TODIM based approach from the sustainability perspective through criteria evaluation is introduced, coping with heterogeneous types of information and reflecting the risk avoidance behavior of decision makers considering dynamic environmental conditions. To the best of the authors’ knowledge, this is the first study on the technology selection problem for wastewater treatment that integrates all aspects of sustainability with the behavioral characteristics of decision makers such as reference dependency, loss avoidance, risk seeking. The fuzzy approach provides a framework to rank the alternatives more scientifically when an emergency decision may be required such as sudden population changes, land and energy restrictions. The contributions of this study can be summarized as follows: (1) A fuzzy TODIM method based on α-cuts set performed by trapezoidal fuzzy numbers is proposed which provides a reliable way to uncover the interaction of criteria in a dynamic environment by changing qualitative and quantitative data into fuzzy information contrary to traditional approaches. (2) The proposed hybrid methodology in contrast to the conventional single methodologies, represents a rational approach to selecting the optimal technology for WWTPs, which inserts risk-averse behavior of DMs into the decision-making model. (3) This decision model based on fuzzy approach reduces information loss and eliminates biased data to make the decision closer to the real case through the strength of Prospect Theory’s membership function.

## Materials and methods

### Study area and evaluation of alternatives

Istanbul is Turkey's most populous metropolitan city with a population of over 15 million residents, settled on an area of 5.34 km^2^. Being a mega city, Istanbul forms the highest population density in Europe. Due to the fact that it receives a large in-migration, in recent years population growth was recorded as almost twice as expected. Istanbul faced problems in terms of water availability throughout its history, but the situation has worsened with the rapid population growth over the last decade^29^. Wastewater in Turkey, which has not been valued until recently, is nowadays being considered as a possible '*new*' source of clean water to be used especially for non-potable purposes. Due to this reason, technology selection for wastewater treatment process needs to be evaluated to serve not only to meet discharge limits, but also the other aspects related with sustainability such as reuse of treated wastewater and protection of water resources. In Turkey, ecological issues related sustainability including treated wastewater reuse, energy efficiency, and renewable energy usage in WWTP, green house effects, and sludge treatment techniques are not always taken into consideration at the same time rationally. The case study in Istanbul involves four types of technologies applied for four WWTPs with a capacity of more than 100,000 m^3^/day. In this research, the alternatives are collected under four different process titles namely, Conventional Activated Sludge System with pre-clarification and digester (CAS-W/-P) (A1), A2O without pre-clarification (A2O-W/O-P) (A2), 5-stage Bardenpho with pre- clarification (BP-5-W/-P) (A3) and finally, A2O with pre-clarification (A2O-W/-P) (A4).

### Methodology

This study comprise four major parts, in the first part, a hierarchical criteria system for WWT technology selection problem is established, and major criteria and sub-criteria is explained. In the second part, data transformation and normalization is performed using TrFNs. Followed by, criteria and sub-criteria of subjective judgements are weighted via AHP. In addition, aggregate criteria weights are calculated by a linear weighting method from obtained quantitative, and *normalized* qualitative data by deriving from the methodology developed by Abdel-Kader and Dugdale in 2001^20^. In the final part, alternatives of WWT technologies are ranked according to fuzzy TODIM method based on α-cuts set adapting the value function of prospect theory. The development of the decision-making model is performed by MATLAB software and data transformation and normalization and sensitivity analysis with respect to the changing attenuation factor $(\theta )$ and comparative study using methods in the literature.

### Establishment of the criteria system

**Figure 1:**
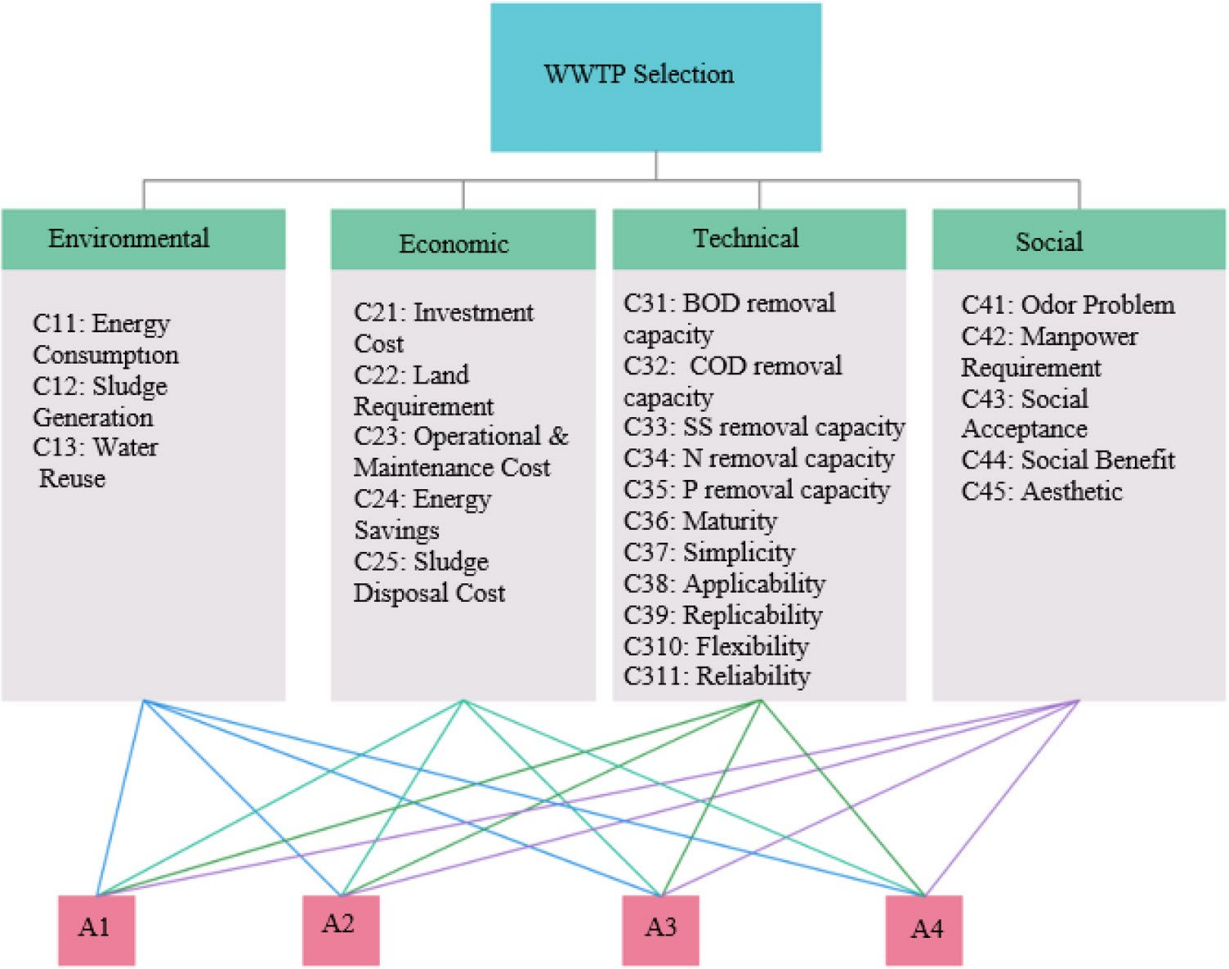
Establishment of criteria system for WWTP selection.

Wastewater treatment activities are considered high-priced and effort-driven due to land requirement, complex processes, and energy cost^30^. Due to this reason, criteria evaluation is considered to be a strategic part of decision making problems considering sustainability^31^. In this study, the dimensions of sustainability were selected based on the following four major criteria: Environmental, economic, technical and social aspects. In order to propose a decision model with criteria evaluation based on scientific foundations, questionnaire study was conducted with experts involved in every stage from the feasibility to construction and operation of WWTPs. Furthermore, environmental impact assessment reports, specifications including design parameters for WWTPs, regulations regarding treated water discharge parameters, and literature were investigated to build a criteria system. Finally, 24 sub-criteria grouped under the main aspects of sustainability based on relevant literature and research group meetings were conducted to build a criteria system that considers the complex process of wastewater treatment as shown in Fig. 1. The definition and explanation of these sub-criteria are shown in Table 1.

**Table 1:** Evaluation criteria for WWTP selection.

Criterion	Explanation
**C1: Environmental criteria**	
C11: Energy consumption	Energy consumption amount during operation to assess carbon footprint, greenhouse gas effect and carbon emissions
C12: Sludge generation	Sludge production of the system
C13: Water reuse	The usage potential of water after treatment
**C2: Economic criteria**	
C21: Investment cost	Expenses for the construction of the wastewater treatment plant
C22: Land requirement	Sufficient space for wastewater treatment plant/future expansion
C23: Operational and maintenance cost	Repair, personnel, chemical and energy costs to manage wastewater treatment plant
C24: Energy savings	Energy recovery to reduce total energy costs of the system
C25: Sludge disposal cost	Expenses of sludge treatment; Sludge Disposal Cost = unit cost of sludge disposal × sludge generation (kg/year)
**C3: Technical criteria**	
C31: BOD removal capacity	The removal capacity of the amount of oxygen consumed by microorganisms while decomposing organic matter
C32: COD removal capacity	The removal capacity of the amount of oxygen consumed to oxidize all organic material by chemical oxidants
C33: SS removal capacity	The removal capacity of the amount of tiny solid particles that act as a colloid
C34: N removal capacity	Nitrogen removal capacity
C35: P removal capacity	Phosphorus removal capacity
C36: Maturity	Applicability of the system
C37: Simplicity	Ease of installation, less operation and maintenance and technical staff requirement, availability of on-line plant tracking
C38: Applicability/operability	Applicable of the system for changing climatic and geographic conditions and population
C39: Replicability	Adoptability to any location under the same conditions to allow practical selection for DMs
C310: Flexibility	Sensitive to changing organic load, hydraulic load, allow process changes
C311: Reliability	Includes plant performance, equipment reliability and emergency operations
**C4: Social criteria**	
C41: Odor problem	Undesired smell potential of the system
C42: Employment	Employment creation of the system
C43: Social acceptance	Public awareness
C44: Social benefit	Added-value of local community to aware environmental issues
C45: Aesthetic	Acceptability of plant conditions and appearance

The Environmental factor includes the impacts of wastewater treatment activities affecting the ecosystem and resources. Considering the environmental impacts, resource consumption and reusability concepts, three criteria were determined to evaluate the environmental efficiency of WWTPs: energy consumption, sludge production, and reuse of treated water. The Economic factor is a measure of the extent of expenditure from the installation phase of the plant to the operation phase that the system operate without interruption including investment cost, operation and maintenance cost and land requirement^32^. Cost-effective and environmentally friendly design of WWTPs is important for decision-makers to balance environmental compliance with budget constraints^33^. For this reason, five economic indicators, including investment cost, land requirement, operational and maintenance cost, energy savings, and sludge disposal cost were used to assess the economics of the WWTPs. The technical factor indicates the treatment efficiency, performance, and technological validation of the treatment plant to achieve the desired objectives dictated by legislation^34^. The technical criteria directly relate to efficiency of treatment process including removal efficiency of biological oxygen demand (BOD), chemical oxygen demand (COD), suspended solid (SS), nitrogen (N) and phosphorus (P) to perform advanced biological treatment. These sub-criteria show the performance of treatment and the fulfillment of design commitment of WWTP. The efficiency is calculated as follows:

$$\text{Efficiency } = \frac{\text{Influent-effluent}}{\text{Influent}} \times \text{ 100 }\left(\text{\% BOD, COD, SS, N, and P}\right).$$

Moreover, six more criteria were added to measure technical performance, which are maturity, simplicity, applicability, replicability, flexibility, and reliability. The Social factor is related to awareness, cultural acceptance, responsibility, and the human resources components of sustainable development, which measures the socio-economic added value to the overall benefits of wastewater treatment plants^35^.

The criteria assessment is carried out by an expert group consisting of a design engineer, a construction engineer, an operation engineer, and an environmental impact assessment expert. The feedbacks from four experts with the equal knowledge and experience in their discipline are evaluated. In this context, the expert weight vector is defined as $\overline{w }$ $=(0.25, \mathrm{0.25,0.25,0.25})$ and, the linguistic set is defined as *l* = {l_0_ = unimportant, l_1_ = equally important, l_2_ = important, l_3_ = more important, l_4_ = much more important}.

The procedure to determine subjective judgements of each criteria by using AHP are as follows^25^:

i. The pairwise comparison matrix A is established and standardized.
ii. Each column element of the subjective judgements matrix, ${a}_{jk}$ is normalized according to the following equation and normalized pairwise comparison matrix represented as N
  1 $${\overline{a} }_{jk}= \frac{{a}_{jk}}{\sum_{i=1}^{n}{a}_{jk}},\,i\,=\,\mathrm{1,2},\dots ,n\, \mathrm{and }\,j=\mathrm{1,2},\dots ,m$$
  where ${\overline{a} }_{jk}$ is the element of N.
iii. The relative weights of criteria or criteria weight vector are obtained by the row average of normalized matrix N. Eigen vector, ${\overline{w} }_{j}$ is calculated to represent criteria weight vector and expressed as follows.
  2 $${w}_{j}= \sum_{k=1}^{n}{a}_{jk}$$
  3 $${\overline{w} }_{j }= \frac{{w}_{j}}{\sum_{i=1}^{n}{w}_{j}}$$

iv. Final step is calculation of the consistency index (CI) to measure consistency of experts’ judgements according to Eq. (4)
  4 $$CI= \frac{{\lambda }_{max}-n}{n-1},$$
  where ${\lambda }_{max}$ is the maximum eigenvalue, n is the rank of the pairwise comparison matrix and $CI<0.1$ is acceptable for the consistency of the pairwise matrix.

**Figure 2:**
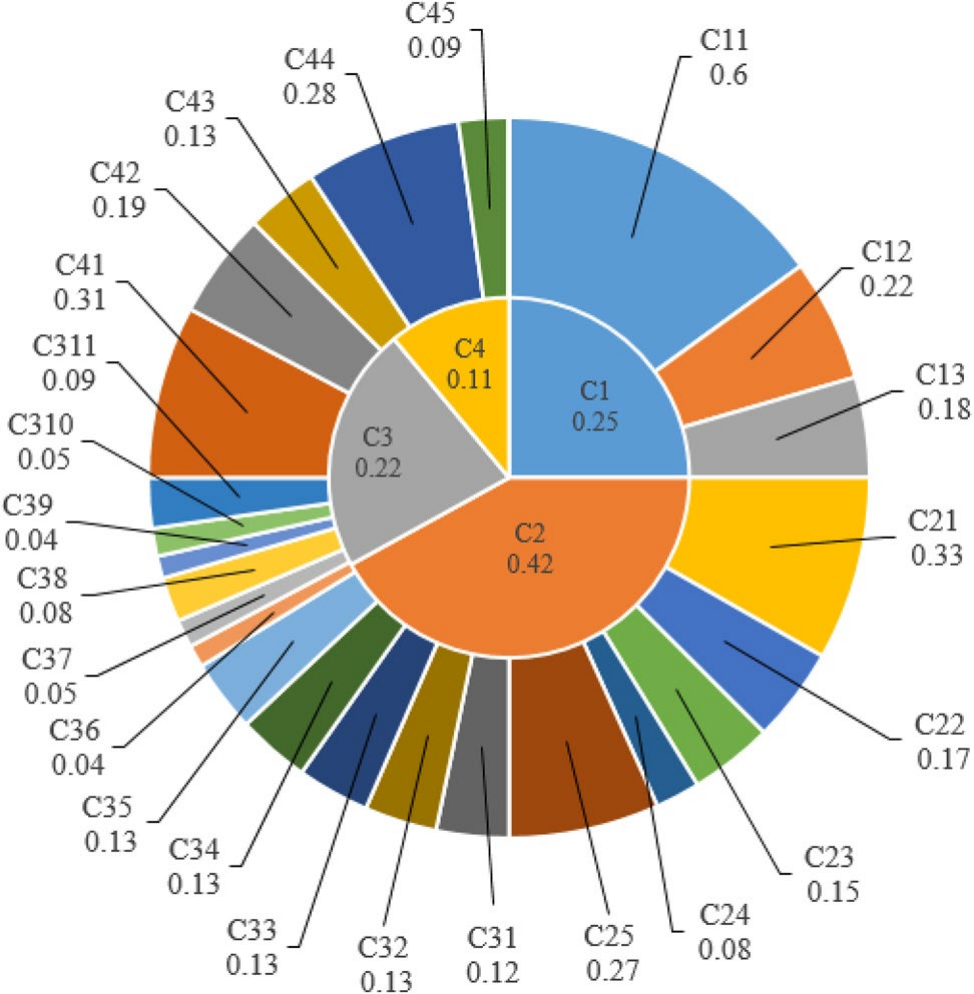
Weights of criteria and sub-criteria.

The criteria weights obtained from AHP are shown in Fig. 2. According to the assessments, economic factors (C2) are of primary importance and the effect of sludge disposal cost (C25) as a sub-criterion appears to be higher than other economic indicators. In order to make a weighting that can cope with the uncertainty arising from subjective judgments or inaccurate data in decision problems containing qualitative and quantitative data, the criterion weights to be included in the decision model were determined by using TrFNs at the second phase of this study.

### Trapezoidal fuzzy numbers

The simplification of a fuzzy number is effectively achieved by the piecewise linear curves that results in a triangular, trapezoidal, or orthogonal membership function^36^. In this study TrFN is used to model fuzzy data for reasons of TrFN’s effectiveness in solving MCDM problems where the lack of knowledge, and ambiguity of human decision-making process exists^37^. On the other hand, there are studies that suggest that TrFN is capable of modelling inaccuracies and reflecting the ambiguous nature of subjective judgments^38,39^.

#### Definition 1

A TrFN $\tilde{a }$ is a special fuzzy subset on the real number set represented as $\tilde{a }=({a}_{1}, {a}_{2},{a}_{3},{a}_{4})$ whose membership function is as follows^36^.

5 $${\mu }_{\tilde{a }} (x)=\left\{\begin{array}{c}0, \,x<{a}_{1}\\ \frac{X-{a}_{1}}{{a}_{2}-{a}_{1}}, \,{a}_{2}\ge x \ge {a}_{1}\\ 1,\, { a}_{3}\ge x \ge {a}_{2}\\ \frac{X-{a}_{3}}{{a}_{3}-{a}_{4}}, { a}_{4}\ge x \ge {a}_{3}\\ 0, \,x>{a}_{4},\end{array}\right.$$

where the ${a}_{1}$ and ${a}_{4}$ are lower and upper limits of $\tilde{a }$ respectively and $[{a}_{2}$, ${a}_{3}]$ is a closed interval.

#### Definition 2

Widely used standard method for defuzzification is the centroid method*.* S ($\tilde{a })$ represents defuzzified value of a trapezoidal fuzzy number calculated as follows^40^:

6 $$S \left(\tilde{a }\right)=\frac{{a}_{1}+ {a}_{2}+{a}_{3}+{a}_{4}}{4}.$$

#### Definition 3

Let $\tilde{a }$ and $\tilde{b }$ be two trapezoidal fuzzy numbers such that, $\tilde{a }=({a}_{1}, {a}_{2},{a}_{3},{a}_{4}$) and $\tilde{b }=\left({b}_{1}, {b}_{2},{b}_{3},{b}_{4}\right).$ Then Euclidean distance $d\left(\tilde{a }, \tilde{b }\right)$ between two TrFNs is calculated as^26^:

7 $$d\left( {\tilde{a}, \tilde{b}} \right) = \sqrt {\frac{1}{6}\left[ {\mathop \sum \limits_{i = 1}^{4} \left( {b_{i} - a_{i} } \right)^{2} + \mathop \sum \limits_{{i \in \left\{ {1,\left. 3 \right\}} \right.}} \left( {b_{i} - a_{i} } \right)\left( {b_{i + 1} - a_{i + 1} } \right)} \right]} .$$

### α-Cut into TrFN Sets

The α-cut sets are the way of comparing or ranking the fuzzy numbers without membership functions^41^.

#### Definition 4

Let *Ã* fuzzy number and its α-cuts *Ã*_*α*_ are defined as^42^.

8 $$\begin{aligned} \tilde{A}_{\alpha } & = \left\{ {x \in X {\mid }\mu_{{\tilde{\ A}}} \left( x \right) \ge \alpha } \right. \\ & = \left[ {min\left\{ {x \in X\mu_{{\tilde{\ A}}} \left( x \right) \ge \alpha } \right\},\max \left\{ {x \in X\mu_{{\tilde{\ A}}} \left( x \right) \ge \alpha } \right\}} \right] \\ & = [\left( x \right)_{\alpha }^{L} , \left( x \right)_{\alpha }^{U} ], \\ \end{aligned}$$

where ${\mu }_{\stackrel{\sim }{\tilde{A} }} (x)$ is the membership function of *Ã*, $x\in X$ denotes the elements belonginto the universal set and $\forall \alpha \in [\mathrm{0,1}]$. Each α-cut set consists of closed intervals containing the upper and lower bound values derived from the fuzzy numbers. According to Definition 4, TrFNs are expressed at α-cuts as follows^43^.

$\tilde{A} =({a}_{1}, {a}_{2},{a}_{3},{a}_{4})$ is a TrFN and its α-cut set can be denoted as

9 $${\tilde{\text{A}} }{\alpha }= [{\tilde{A} }_{\alpha }^{L},{\tilde{A} }_{\alpha }^{U}]=\left[{a}_{1}+\alpha \left({a}_{2}-{a}_{1), }{{a}_{4}-\alpha (a}_{4-}{a}_{3}\right)\right].$$

### Prospect theory

The major concept of prospect theory developed by Kahneman and Tversky in 1979^16^ is that decision making depends on behavioral tendency under risks considering the potential value losses and gains representing variability with respect to reference point selection. The value function of prospect theory is described as follows.

10 $$\mu \left(z\right)= \left\{\begin{array}{c}{z}^{\alpha }\, if \quad z \ge 0\\ -\lambda {\left(-z\right)}^{\beta }\, if \quad z<0,\end{array}\right.$$

where z denotes gains or losses; $z \ge 0$ represents the gains and $z<0$ represents losses, and α is the risk seeking coefficient and β is the risk averse coefficient. The expression λ is called the risk aversion coefficient and $\lambda >1$ represents that decision maker is more sensitive to losses than gains. The value function of Prospect Theory is an s-shaped (sigmoidal) function, consisting of concave and convex part representing gains and losses respectively. In summary, decision makers are risk averse for gains and risk-seeking for losses^44^.

Kahneman and Tversky in 1979^16^ defined α, β and λ in their empirical research and determined their values as $\alpha = \beta =0.88$ and, λ = 2.25.

### The TODIM method

The TODIM Method is attributed as a hybrid method combining aspect of MAUT (Multiattribute Utility Theory) of AHP and ELECTRE methods^17^. In general, MCDM problems have a set of finite number of alternatives: $A= \left\{{A}_{1}, \dots , {A}_{m}\right\}$ and a set of finite number of criteria: $C= \left\{{C}_{1}, \dots , {C}_{n}\right\}.$ The weighting vector of criteria $W= \left\{{w}_{1}, \dots , {w}_{n}\right\}$ and the individual weight ${w}_{k}$; $k=\left\{1, \dots , n\right\}$ for each criterion ${C}_{k}$ satisfying $\sum_{k=1}^{n} {w}_{k}=1$. Let $D={({x}_{ik})}_{mxn}$ be a normalized decision matrix ${x}_{ik}$ denoting the assessment or performance of alternative ${A}_{i}$ related to criterion ${C}_{k}$ in the form of crisp number with $i\in M,$ where $M= \left\{1,..,m\right\}$, $k\in N$, and $N= \left\{1,..,n\right\}$. The decision making procedure for TODIM method is described as follows:

**Step 1:** Calculate the relative weight ${w}_{kr}$ of criterion ${C}_{k}$ to the reference criterion ${C}_{r}$ as follows:

11 $${w}_{kr}= \frac{{w}_{k}}{{w}_{r}},$$

where ${w}_{k}$ denotes the weight of the criterion ${C}_{k}$ and ${w}_{r}$ = max $\left\{{w}_{k} \left|k \in N)\right.\right\}.$

**Step 2:** TODIM method relies on the dominance of one alternative ($${A}_{i})$$ over another alternative $$\left({A}_{j}\right)$$ under criterion k calculated by using the value function of Prospect Theory expressed following:

12 $${\phi }_{k}\left({A}_{i}, {A}_{j}\right)=\left\{ \begin{array}{c}\sqrt{\left|{x}_{ik}- {x}_{jk}\right| \frac{{w}_{kr}}{\sum_{k=1}^{n}{w}_{kr}}} \quad {x}_{ik}- {x}_{jk}>0\\ 0 \quad {x}_{ik}- {x}_{jk}=0\\ - \frac{1}{\theta }\sqrt{\left|{x}_{ik}- {x}_{jk}\right| \frac{\sum_{k=1}^{n}{w}_{kr}}{{w}_{kr}}} \quad {x}_{ik}- {x}_{jk}< 0,\end{array}\right.$$

where $\theta$ denotes attenuation factor that evaluate loss aversion. θ > 0 represents high risk aversion preference of DM. If θ < 0, less risk aversion or higher risk seeking attribute of DM reflected on ranking alternatives.

**Step 3:** Obtain the overall dominance degree of each alternative ${A}_{i}$ over each ${A}_{j}$ calculated by:

13 $$\phi \left({A}_{i}, {A}_{j} \right)= \sum_{k=1}^{n}{\phi }_{k}\left({A}_{i}, {A}_{j} \right) i,j \in M \& k\in N.$$

**Step 4:** Calculate the global dominance of alternative ${A}_{i}$ as follows:

14 $${\xi \left({A}_{i}\right)}_{= }\frac{\sum_{j=1}^{m}\phi \left({A}_{i}, {A}_{j} \right)-\mathrm{min}\sum_{j=1}^{m}\phi \left({A}_{i}, {A}_{j} \right)}{\mathrm{max}\sum_{j=1}^{m}\phi \left({A}_{i}, {A}_{j} \right)- \mathrm{min}\sum_{j=1}^{m}\phi \left({A}_{i}, {A}_{j} \right)} i\in M.$$

**Step 5:** Calculation of the global dominance of each alternative makes possible to rank of alternatives. The alternative has higher value of $\xi$ is the best alternative.

### Decision-making process: fuzzy TODIM based on α-cuts to select WWTP

**Figure 3:**
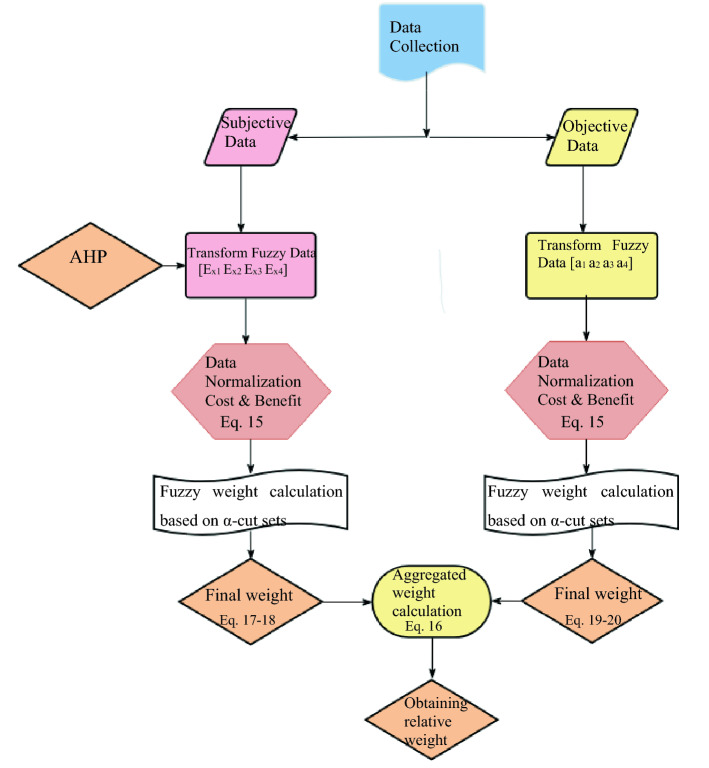
Creation of the weighted decision matrix.

**Figure 4:**
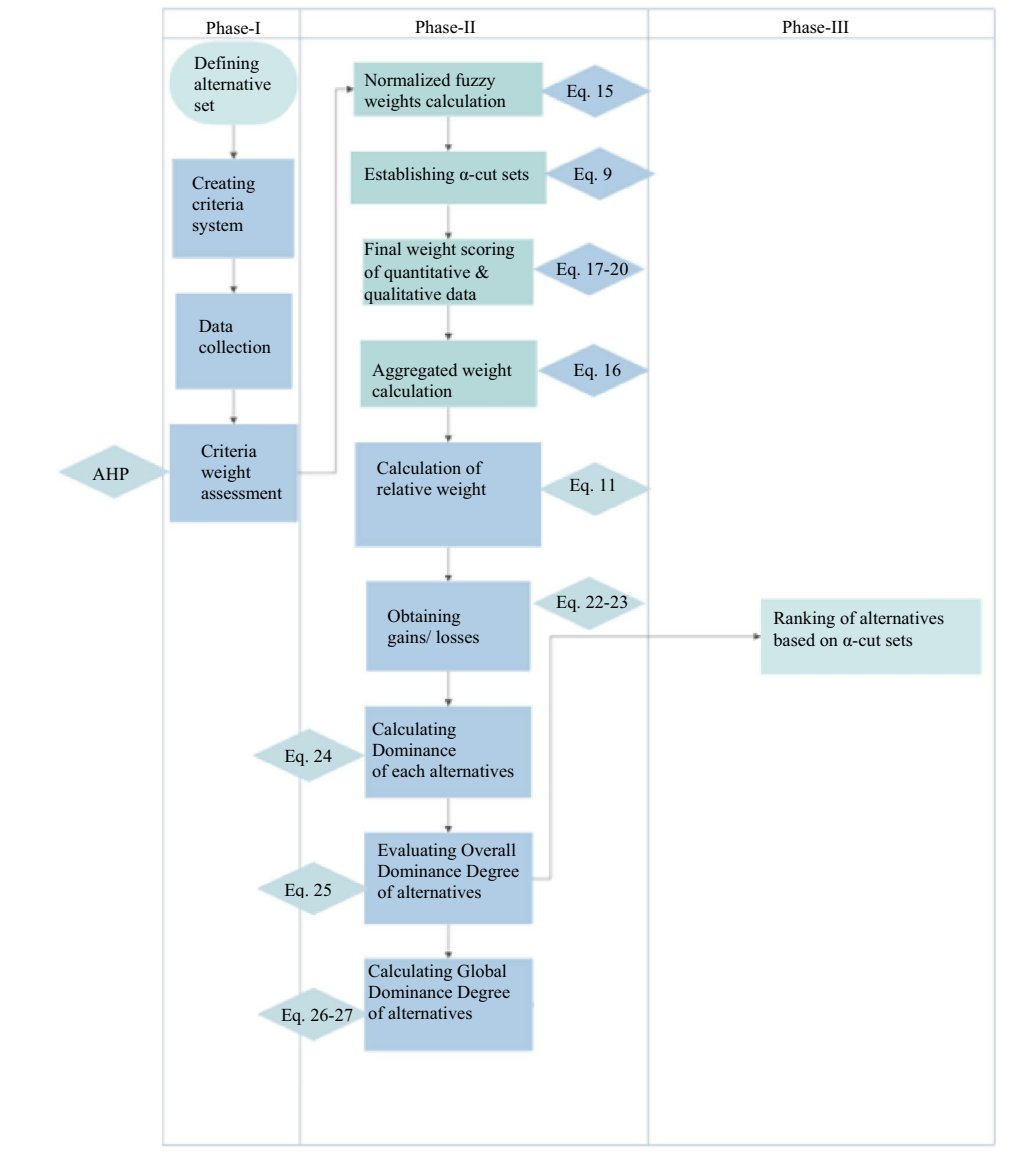
Decision making process in this study.

Since the classic MCDM tools do not take into account the risk orientations of the decision-makers, the situation arises that the model is insufficient in the face of dynamic changes^27^. Unforeseen parameters such as sudden population changes, catastrophes, unfavorable conditions due to climate change, lack of space, energy costs lead to the fact that the phenomenon that manages the uncertainty is chosen according to the behavior of risk avoidance and not loss avoidance. The real-form of this behavior expressed as a function is the prospect theory membership function. TODIM is a technique derived from Prospect Theory that puts the membership function of Prospect Theory at the center of the decision making process to reflect the rational behavior of DMs^45^. In this context, the framework for the decision-making process includes the weighting to obtain relative weights of criteria is retrieved from Fig. 3 and consists of three phases as can be seen in Fig. 4. The section on determining relative weights, shown in Fig. 3, begins with the collection of subjective and objective data. The experts’ opinion as subjective data evaluated using AHP by transformation of TrFNs in relation to different expert perspective and, real-scale operation information belongs to WWTPs as objective data is transformed TrFNs mentioned in “Data transformation” section. The weighted criteria require a normalization procedure for final weights depending on alpha cut series to produce compromise solutions. At the end of the weighting procedure, both subjective and objective weights are aggregated to calculate the dominance degree of the alternatives. The aggregated weights are inserted into the membership function of TODIM to rank alternatives. The detailed decision-making procedure is also explained follow accompanied by Fig. 4.

Phase-I: Figuration includes (a) defining an alternative sets (b) establishment of criteria system with respect to available technology of WWTP processing, (c) data collection from design engineer, construction engineer, process or operation engineer and environmental impact assessment expert, (d) priority evaluation of criteria and sub-criteria in terms of weight calculation through AHP.

Phase-II: Modelling and Evaluation composes of (a) data normalization procedure of TrFNs, (b) weighting of subjective judgements and objective data based on *α-cuts* and derived the membership function, (c) calculation of aggregate weights of criteria, (d) converting TrFNs into their upper and lower bounds to obtain their *α-cuts* for crisp data (e) obtaining the gains and losses, (f) calculating dominance of each alternatives based on the gains or losses, (g) evaluating overall dominance degree of alternatives, (h) calculating global dominance of alternatives.

Phase III: Selection encloses ranking of alternatives according to its global dominance degree at each alpha level sets. A solution environment will be provided to DMs in which they can evaluate the most compromised solution according to global dominance degree at each alpha level sets. In addition, presenting the average global dominance degrees considering alpha cuts guide DMs to choose the most consistent solution for process of WWTP.

### Data transformation

In order to, reflect the ambiguous nature of subjective judgements and uncertainty of inaccurate information, trapezoidal fuzzy numbers were used in decision model. Under the condition of symmetric uncertainty around the mean, crisp and linguistic data were changed into TrFNs. 20% of uncertainty to compose with trapezoidal fuzzy numbers and %5 of symmetric uncertainty around the mean calculated as follows: ${a}_{1}=t-0.2 t, {a}_{2}= t-0.05 t, { a}_{3 =}t+0.05 t, {a}_{4=} t+0.2 t$ where *t* denotes the collected crisp data value. Additionally, linguistic terms are transformed to TrFNs via their mapping relations showed in Table 2. Linguistic terms are determined by survey of wastewater treatment plant’s operators and experts with respect to 1–10 scale.

**Table 2:** Linguistic terms and their expression based on TrFNs.

Linguistic terms	1–10 scale
Extremely poor (EP)	1
Very poor (VP)	2
Fairly poor (FP)	3
Poor (P)	4
Medium (M)	5
Fairly strong (FS)	6
Strong (S)	7
Very strong (VS)	8
Extremely strong (ES)	9,10

	Trapezoidal interval fuzzy number
EP	(0.8, 0.95, 1.05, 1,2)
VP	(1.6, 1.9, 2.1, 2.4)
FP	(2.4, 2.85, 3.15, 3.6)
P	(3.2, 3.8, 4.2, 4.8)
M	(4.0, 4.75, 5.25, 6.0)
FS	(4.8, 5.7, 6.3, 7.2)
VS	(5.6, 6.65, 7.35, 8.4)
ES	(6.4, 7.6, 8.4, 9.6)
	(7.2, 8.55, 9.45, 9.6)
	(8.0, 9.5, 10.5, 12,0)

### Data normalization

To obtain normalized decision matrix, assume that there is an alternative set of $A= \left\{{A}_{1}, \dots , {A}_{m}\right\}$ including m alternatives, a criteria set of $C= \left\{{C}_{1}, \dots , {C}_{n}\right\}$ including n criteria, and the fuzzy decision matrix $\tilde{A }=[{{\tilde{x }}_{ij}]}_{m x n}$ where ${\tilde{x }}_{ij}$ denotes the information of alternative ${A}_{i}$ related to criterion ${C}_{j}$ transformed into TrFNs. Then, the fuzzy decision matrix is converted into the normalized decision matrix as $\tilde{A }$ $=[{{\tilde{r }}_{ij}]}_{m \times n}$.

The criteria are separated into two groups benefit and cost criteria. The normalization of criteria is performed by:

$${\tilde{r }}_{ij}^{k}= \frac{\mathrm{max}\left({a}_{ij}^{4}\right)- {a}_{ij}^{k}}{{max}_{i}\left({a}_{ij}^{4}\right)-{min}_{i}({a}_{ij}^{1})} \quad k=\mathrm{1,2},\mathrm{3,4 }{x}_{ij }\in {F}^{C},$$

15 $${\tilde{r }}_{ij}^{k}\,= \,\frac{{a}_{\mathit{ij} }^{k}- {min}_{i}({a}_{ij}^{1})}{{max}_{i}\left({a}_{ij}^{4}\right)- {min}_{i}({a}_{ij}^{1})}\quad k\,=\,\mathrm{1,2},\,\mathrm{3,4} {x}_{ij }\in {F}^{B}.$$

### Determination of criteria weights

In the decision model presented in this study, an aggregate weighting approach is proposed in order to include both subjective judgements and objective data. During the operation phase of wastewater treatment plants, both quantitative data and engineering experiences are taken into consideration while evaluating plant efficiency. The methodology is developed by Abdel-Kader and Dugdale^20^ are employed to calculate final weight scoring of subjective judgement and objective data.

The aggregated weight is calculated to obtain holistic approach for weighting by linear weighting method as follows:

16 $${w}_{j\_agg}= \gamma {w}_{j\_sub}+\lambda {w}_{j\_ob} \quad \gamma +\lambda =1,\, and \,\gamma , \lambda >0,$$

where $w{j}_{agg}$, ${w}_{j\_sub}$, and ${w}_{j\_ob}$ are denoted aggregated weight, subjective weight and objective weight vectors respectively. In this study, γ and λ represents the influence coefficient of the subjective and objective weights, respectively which were assumed to be 0.5.

### Determination of subjective weights of criteria

In order to make a realistic criteria evaluation, it is necessary to deal with the ambiguity of subjective judgments and data inconsistencies. Hence, the effect of subjective judgment on the criteria is evaluated in two stages. In the first stage, the first weights of the criteria according to the priority evaluation performed by the experts are determined via AHP. The results of subjective judgements are represented in Table 3. The evaluation results of subjective judgements set is expressed $E=\left\{{E}_{1},\dots , {E}_{t}\right\}$ and, t is the number of experts. A criteria set is defined as $C= \left\{{C}_{1}, \dots , {C}_{n}\right\}$ and, n is the number of criteria. The evaluated results through AHP are converted into the linear fuzzy weights of criteria expressed as ${\tilde{w }}_{E}=[\tilde{w }{]}_{t x n}$. The normalized fuzzy weights are calculated by Eq. (15) according to benefit and cost criteria. As the last phase, to keep uncertain environment as much as possible α-cut sets are employed rather than centroid method. The corresponding normalized fuzzy weights are expressed as α-cuts (α = {0, 0.1, 0.2, 0.3, 0.4, 0.5, 0.6, 0.7, 0.8, 0.9, 1.0}) by Eq. (9) ^46^. In order to determine final weight scoring, a membership function is suggested which is derived from the methodology is developed by Abdel-Kader and Dugdale^20^ to achieve final subjective weight scoring. The methodology is used in fuzzy multi-criteria decision making models based on α-cuts considering risk seeking or risk avoiding perspective of DMs which enables to compare solution set for compromise solutions^47^. The membership function for final weighting is as follows:

$${\mu }_{s}({w}_{k}{)}_{\alpha }=\left[\beta \left(\frac{{\left({\tilde{x }}_{k}\right)}_{\alpha }^{U}-\mathrm{min}({{\tilde{x }}_{k})}^{L}}{\mathrm{max}({{\tilde{x }}_{k})}^{U}-\mathrm{min}({{\tilde{x }}_{k})}^{L}+{\left({\tilde{x }}_{k}\right)}_{\alpha }^{U}-{\left({\tilde{x }}_{k}\right)}_{\alpha }^{L}}\right)\right.$$

17 $$+\left.(1-\beta )\left( 1-\frac{\mathrm{max}({{\tilde{x }}_{k})}^{U}-{\left({\tilde{x }}_{k}\right)}_{\alpha }^{L}}{\mathrm{max}({{\tilde{x }}_{k})}^{U}-\mathrm{min}({{\tilde{x }}_{k})}^{L}+{\left({\tilde{x }}_{k}\right)}_{\alpha }^{U}-{\left({\tilde{x }}_{k}\right)}_{\alpha }^{L}}\right)\right] \left[\frac{{\left({\tilde{x }}_{k}\right)}_{\alpha }^{U}+{\left({\tilde{x }}_{k}\right)}_{\alpha }^{L}}{2}\right],$$

where ${\left(\tilde{x }\right)}_{\alpha },$= [${\left(x\right)}_{\alpha }^{L}, {\left(x\right)}_{\alpha }^{U}]$ is the α-cuts of $\tilde{x }$ which denotes subjective criteria weights. The $\mathrm{max}({{\tilde{x }}_{k})}^{U}$ and, $\mathrm{min}({{\tilde{x }}_{k})}^{L}$ shows the value of the maximum upper, and minimum lower bound of $\tilde{x }$ related to criterion k among eleven alpha level sets (α-cuts) respectively. In the derived membership function, $\beta$ represents the risk tendencies of the decision makers (risk averse or risk seeking) and its value defined as 0.5. The calculated weights can be normalized as follows:

**Table 3:** Evaluation results of subjective judgements through AHP.

Criteria	Expert-1	Expert-2	Expert-3	Expert-4
C11	0.21	0.49	0.40	0.26
C12	0.55	0.31	0.40	0.41
C13	0.24	0.19	0.20	0.33
C21	0.24	0.29	0.09	0.14
C22	0.25	0.34	0.11	0.21
C23	0.22	0.12	0.28	0.21
C24	0.13	0.12	0.26	0.14
C25	0.16	0.11	0.26	0.30
C31	0.13	0.12	0.15	0.17
C32	0.13	0.12	0.15	0.17
C33	0.13	0.12	0.15	0.17
C34	0.13	0.12	0.15	0.17
C35	0.13	0.12	0.15	0.17
C36	0.04	0.02	0.02	0.01
C37	0.05	0.04	0.04	0.01
C38	0.08	0.11	0.07	0.03
C39	0.05	0.06	0.03	0.01
C310	0.04	0.07	0.02	0.01
C311	0.09	0.10	0.07	0.08
C41	0.27	0.14	0.24	0.36
C42	0.16	0.18	0.18	0.10
C43	0.17	0.25	0.24	0.22
C44	0.20	0.18	0.23	0.22
C45	0.20	0.25	0.11	0.10
CI_11-13_	2.0%	6.0%	0.0%	6.0%
CI_21-25_	8.9%	1.0%	1.0%	7.0%
CI_31-311_	6.0%	1.0%	2.0%	1.0%
CI_41-45_	2.0%	5.0%	8.0%	2.0%

18 $$({w}_{k}{)}_{\alpha }= \frac{({w}_{k}{)}_{\alpha }}{\sum_{k=1}^{n}({w}_{k}{)}_{\alpha }}.$$

The results of normalized weights matrix of evaluated subjective judgements are represented in Supplementary Information section.

### Determination of objective weights of criteria

The evaluation of quantitative data gathering from operation department of WWTPs includes data transformation, normalization and, obtaining objective criteria weights phases. The approach to converting crisp values to TrFNs and normalization procedure is discussed in “Data transformation” and “Data normalization”, respectively.

As a final scoring of quantitative data to calculate compromise weighting vector based on α-cut sets Eqs. (8) and (9) are employed with respect to eleven alpha levels, α = $\left\{0, 0.1, 0.2, 0.3, 0.4, 0.5, 0.6, 0.7, 0.8, 0.9, 1.0\right\}$. To obtain the decision weighting vector of quantitative data, the membership function and its normalized form is as follows:

$${\mu }_{o}({w}_{ik}{)}_{\alpha }=\left[\beta \left(\frac{{\left({\tilde{x }}_{ik}\right)}_{\alpha }^{U}-\mathrm{min}({{\tilde{x }}_{ik})}^{L}}{\mathrm{max}({{\tilde{x }}_{ik})}^{U}-\mathrm{min}({{\tilde{x }}_{ik})}^{L}+{\left({\tilde{x }}_{ik}\right)}_{\alpha }^{U}-{\left({\tilde{x }}_{ik}\right)}_{\alpha }^{L}}\right)\right.$$

19 $$+\left.(1-\beta )\left( 1-\frac{\mathrm{max}({{\tilde{xi }}_{k})}^{U}-{\left({\tilde{x }i}_{k}\right)}_{\alpha }^{L}}{\mathrm{max}({{\tilde{x }}_{ik})}^{U}-\mathrm{min}({{\tilde{x }}_{ik})}^{L}+{\left({\tilde{x }}_{ik}\right)}_{\alpha }^{U}-{\left({\tilde{xi }}_{k}\right)}_{\alpha }^{L}}\right)\right] \left[\frac{{\left({\tilde{x }}_{ik}\right)}_{\alpha }^{U}+{\left({\tilde{xi }}_{k}\right)}_{\alpha }^{L}}{2}\right],$$

where ${\left(\tilde{x }\right)}_{\alpha },$= [${\left({\tilde{x }}_{ik}\right)}_{\alpha }^{L}, {\left({\tilde{x }}_{ik}\right)}_{\alpha }^{U}]$ is the α-cuts of $\tilde{x }$ which denotes quantitative criteria weights. The $\mathrm{max}({{\tilde{x }}_{ik})}^{U}$ and, $\mathrm{min}({{\tilde{x }}_{ik})}^{L}$ shows the value of the maximum upper, and minimum lower bound of ${\tilde{x }}_{i}$ represented performance of alternative ${A}_{i}$ related to criterion k among eleven alpha level sets (α-cuts) respectively. In the derived membership function, $\beta$ represents the risk behavior of the decision makers (risk averse or risk seeking) and its value defined as 0.5. The calculated weights can be normalized as follows:

20 $$({w}_{k}{)}_{\alpha }= \frac{({w}_{k}{)}_{\alpha }}{\sum_{k=1}^{n}({w}_{k}{)}_{\alpha }}.$$

The results of normalized weights of quantitative data are available from Supplementary Information section.

### Dominance of each alternative

The fuzzy TODIM approach is implemented with the fuzzy criterion weights and the distance calculation between two fuzzy numbers to obtain overall dominance degree of each alternative. Dominance of each alternative ${\tilde{A }}_{i}$ over each alternative ${\tilde{A }}_{j}$ is calculated by the membership function of Prospect Theory is based on gains or losses performed by trapezoidal fuzzy numbers to evaluate risk aversion degree in the presence of uncertain environment as follows.

21 $${\varphi }_{k}\left({\tilde{A }}_{i}, \tilde{A }_{j}\right)=\left\{\begin{array}{c} \sqrt{ d\left({\tilde{x }}_{ik},\,{\tilde{x }}_{jk}\right) \frac{{w}_{kr}}{\sum_{k=1}^{n}{w}_{kr}}}\quad if \quad \left[S \left({\tilde{x }}_{ik}\right)-S \left({\tilde{x }}_{jk}\right)\right]>0, \\ 0\quad if \quad \left[S \left({\tilde{x }}_{ik}\right)-S \left({\tilde{x }}_{jk}\right)\right]=0, and\\ - \frac{1}{\theta }\sqrt{ d\left({\tilde{x }}_{ik},\,{\tilde{x }}_{jk}\right) \frac{\sum_{k=1}^{n}{w}_{kr}}{{w}_{kr}}} \quad if \quad \left[S \left({\tilde{x }}_{ik}\right)-S \left({\tilde{x }}_{jk}\right)\right]<0. \end{array}\right.$$

$S \left({\tilde{x }}_{ik}\right)$ and $S \left({\tilde{x }}_{jk}\right)$ parameters are defuzzied values which allow to compare two trapezoidal fuzzy numbers for construction of final decision matrix according to defuzzification function expressed as $S \left({\tilde{x }}_{ik}\right)-S \left({\tilde{x }}_{jk}\right)$. The expression $d\left({\tilde{x }}_{ik},{\tilde{x }}_{jk}\right)$ is the distance between two trapezoidal fuzzy numbers. The defuzzification function employs to determine gain, loss or nil conditions. The use of the distance, $d\left({\tilde{x }}_{ik},{\tilde{x }}_{jk}\right),$ to calculate dominance degree rather than defuzzification function appears logical, since the property $0\le d\left({\tilde{x }}_{ik},{\tilde{x }}_{jk}\right)\le 1$ is satisfied. Three conditions are available in terms of gain or loss presented below. Gains, losses and nil are fitted into the membership function of Cumulative Prospect Theory as anticipated. The expression of ${\varphi }_{k}$ emphasizes contribution of the criterion k to function $\delta \left({\tilde{A }}_{i}, {\tilde{A}}_{j}\right)$ when comparing the alternative i with j. $\theta$ denotes the attenuation factor of the loss. The value of $\theta$ should satisfy the condition $\theta >0$, which indicates the degree of experts’ loss averse preference. If $0<\theta <1$, then the impact of loss increases, if $\theta >1$, the impact of loss decreases^48^.

For attenuation factor $\theta$ which is taken 2.25 in this proposed decision framework:

i. $$S \left({\tilde{x }}_{ik}\right)$$ − $$S \left({\tilde{x }}_{jk}\right)$$> 0; gain
ii. $$S \left({\tilde{x }}_{ik}\right) - S \left({\tilde{x }}_{jk}\right)$$ = 0; nil
iii. $$S \left({\tilde{x }}_{ik}\right) - S \left({\tilde{x }}_{jk}\right)$$ < 0; loss

The fuzzy TODIM based on α-cuts calculate dominance of each alternative according to different α-cuts of TrFNs and relative weights of criteria considering alpha level sets. To determine dominance degree of alternatives based on α-cut sets, gains and losses are calculated by upper and lower bounds of TrFNs instead of defuzzied values. On the other hand, for reliable and compromise ranking of alternative according to dominance degree is performed by interval of dominance degree rather than the distance calculation of two TrFNs. In order to realistic evaluation of criteria and expected alternative ranking fuzzy conditions should be preserved as much as possible in the decision process model. The α-cut are the effective forms of fuzzy sets to cope with uncertain environment along with decision process^49^. The expression of (${{\tilde{x }}_{ik})}_{\alpha }$ and (${{\tilde{x }}_{jk})}_{\alpha }$ are two intervals of TrFNs which are denoted as (${{\tilde{x }}_{ik})}_{\alpha }=\left[{({\tilde{x }}_{ik})}_{\alpha }^{L}, {({\tilde{x }}_{ik})}_{\alpha }^{U}\right]$ and (${{\tilde{x }}_{jk})}_{\alpha }=\left[{({\tilde{x }}_{jk})}_{\alpha }^{L}, {({\tilde{x }}_{jk})}_{\alpha }^{U}\right]$. The dominance degree of (${{\tilde{x }}_{ik})}_{\alpha }$ over (${{\tilde{x }}_{jk})}_{\alpha }$ are acquired by Ref.^50^ is calculated by:

22 $$\mathrm{P }(({{\tilde{x }}_{ik})}_{\alpha }>({{\tilde{x }}_{jk})}_{\alpha })\,=\,\frac{max\left[0, {\left({\tilde{x }}_{\dot{\text{i}}k}\right)}_{\alpha }^{U}-{\left({\tilde{x }}_{jk}\right)}_{\alpha }^{L}\right]-max\left[0, {\left({\tilde{x }}_{\dot{\text{i}}k}\right)}_{\alpha }^{L}-{\left({\tilde{x }}_{jk}\right)}_{\alpha }^{U}\right]}{\left[ {\left({\tilde{x }}_{\dot{\text{i}}k}\right)}_{\alpha }^{U}-{\left({\tilde{x }}_{\dot{\text{i}}k}\right)}_{\alpha }^{L}\right]+\left[ {\left({\tilde{x }}_{jk}\right)}_{\alpha }^{U}-{\left({\tilde{x }}_{jk}\right)}_{\alpha }^{L}\right]}$$

Gains and losses are expressed according to the following two conditions.

i. $$P (({{\tilde{x }}_{ik})}_{\alpha }>({{\tilde{x }}_{jk})}_{\alpha })\ge P (({{\tilde{x }}_{jk})}_{\alpha }>\left({{\tilde{x }}_{ik})}_{\alpha }\right);$$
  $${\rho }_{\alpha }^{+}(({{\tilde{x }}_{ik})}_{\alpha }>\left({{\tilde{x }}_{jk})}_{\alpha }\right)=P (({{\tilde{x }}_{ik})}_{\alpha }>\left({{\tilde{x }}_{jk})}_{\alpha }\right)-P (({{\tilde{x }}_{jk})}_{\alpha }>\left({{\tilde{x }}_{ik})}_{\alpha }\right)$$
ii. $$P (({{\tilde{x }}_{ik})}_{\alpha }>({{\tilde{x }}_{jk})}_{\alpha })<P (({{\tilde{x }}_{jk})}_{\alpha }>\left({{\tilde{x }}_{ik})}_{\alpha }\right);$$
  23 $${\rho }_{\alpha }^{-}(({{\tilde{x }}_{ik})}_{\alpha }>\left({{\tilde{x }}_{jk})}_{\alpha }\right)=P (({{\tilde{x }}_{jk})}_{\alpha }>\left({{\tilde{x }}_{ik})}_{\alpha }\right)-P (({{\tilde{x }}_{ik})}_{\alpha }>\left({{\tilde{x }}_{jk})}_{\alpha }\right),$$
  where ${\rho }_{\alpha }^{+}(({{\tilde{x }}_{ik})}_{\alpha }>({{\tilde{x }}_{jk})}_{\alpha })$ and ${\rho }_{\alpha }^{-}(({{\tilde{x }}_{ik})}_{\alpha }>({{\tilde{x }}_{jk})}_{\alpha })$ denotes gains and losses respectively.

In this context, the dominance degree of alternative A_i_ over A_j_ with respect to criterion C_k_ based on α-cut sets can be obtained by rearranging as follows.

24 $${\phi }_{k}^{\alpha }\left({A}_{i}, {A}_{j}\right)=\left\{\begin{array}{c}\sqrt{ { \rho }_{\alpha }^{+}(({{\tilde{x }}_{ik})}_{\alpha }>({{\tilde{x }}_{jk})}_{\alpha }) \frac{{w}_{kr}}{\sum_{k=1}^{n}{w}_{kr}}} \; if \quad P (({{\tilde{x }}_{ik})}_{\alpha }>({{\tilde{x }}_{jk})}_{\alpha })\ge P (({{\tilde{x }}_{jk})}_{\alpha }>\left({{\tilde{x }}_{ik})}_{\alpha }\right) \\ \\ - \frac{1}{\theta }\sqrt{ {\rho }_{\alpha }^{-}(({{\tilde{x }}_{ik})}_{\alpha }>({{\tilde{x }}_{jk})}_{\alpha }) \frac{\sum_{k=1}^{n}{w}_{kr}}{{w}_{kr}}} \; if \quad P (({{\tilde{x }}_{ik})}_{\alpha }>({{\tilde{x }}_{jk})}_{\alpha })<P (({{\tilde{x }}_{jk})}_{\alpha }>\left({{\tilde{x }}_{ik})}_{\alpha }\right). \end{array}\right.$$

The overall dominance degree of alternative ${\tilde{A }}_{ \dot{\text{{I}}} }$ over alternative ${\tilde{A }}_{j}$ is necessary to obtain *the global value of alternatives* that is calculated by:

25 $${\delta }_{\alpha }\left({A}_{i}, {A}_{j}\right)= \sum_{k=1}^{n}{\phi }_{k}^{\alpha }\left({A}_{i}, {A}_{j}\right) \forall \left(i,j\right),$$

where ${\delta }_{\alpha }\left({A}_{i}, {A}_{j}\right)$ represents the measurement of overall dominance degree of alternative ${A}_{i}$ over alternative ${A}_{j}$ based on α-cuts, n is the number of criteria; k is any criterion for k = 1, …, n.

### Calculation of global dominance of each alternative

The overall value of alternative i through normalization of the corresponding dominance measurements. The rank of each alternatives are determined the following equation that normalizes the overall dominance degree of alternatives to achieve eleven ranking set.

26 $${\xi }_{\alpha }\left({A}_{i}\right)=\frac{\sum_{j=1}^{m}{\delta }_{\alpha }\left({A}_{i}, {A}_{j}\right) -\mathrm{min}\sum_{j=1}^{m}{\delta }_{\alpha }\left({A}_{i}, {A}_{j}\right) }{\mathrm{max}\sum_{j=1}^{m}{\delta }_{\alpha }\left({A}_{i}, {A}_{j}\right) - \mathrm{min}\sum_{j=1}^{m}{\delta }_{\alpha }\left({A}_{i}, {A}_{j}\right) }.$$

The overall dominance degree calculation based on α-cuts allows to choose the compromise ranking related risk seeking or risk averse choices through solution set. The advantage of the methodology is presented a solution set with respect to attenuation factor, θ to reflect risk perspective of DMs to the decision model and gives the best possible alternative according to the average global dominance degree, ${\xi }_{\alpha }\left({A}_{i}\right)$. The average global dominance degree is calculated by:

27 $$\overline{\xi } \left({A}_{i}\right)= \frac{1}{\beta}{\sum }_{b =1}^{\beta}{\xi }_{{\alpha }_{b} }\left({A}_{i}\right), i=1,\dots , m.$$

### Ethical approval

Ethics committee approval is not required**.**

### Consent to publish

The authors confirm that the final version of the manuscript has been reviewed, approved, and consented for publication by all authors.

## Case study and discussion

### Selection of alternatives

The rapid progress of technological developments in the wastewater sector leads to differentiation of wastewater treatment technologies. Many authorities still prefer mature technologies for a variety of reasons such as lack of qualified personally, risk aversion, or outdated technical information. In Turkey the following treatment technologies are usually applied for WWTPs for plants having capacity of more than 100,000 m^3^/ day (CAS-W/-P or CAS-W/O-P; A2O- W/-P or A2O-W/O-P; BP-5-W/-P or BP-5-W/O-P). In order to solve the possible infrastructure problems that Istanbul may encounter in the near future, high capacity WWTPs should be compared rationally. Based on this need, four types of wastewater treatment plants, which are of critical importance for Istanbul, were evaluated with a decision model, under the guidance of the sustainability concept taking into account the perception of experts. The alternatives are collected under four different process titles namely, CAS-W/-P (A1), A2O-W/O-P (A2), BP-5-W/-P (A3) and A2O W/-P (A4).

Conventional Activated Sludge System includes primary settling, aerobic biological treatment, secondary settling, disinfection, and discharge. It is commonly used as treatment technology for the removal of BOD and COD and, partial nutrient (N-nitrogen and P-phosphorus) removal can also be accomplished. Digester is required for this system since the sludge generated in CAS system is not stable.

A2O without Pre-clarification is a type of activated sludge process where a sequence of anaerobic, anoxic and aerobic tanks/zones are provided to remove organic carbon, nitrogen and phosphorus.

5-stage Bardenpho with Pre-clarification is an A2O process followed by a second anoxic zone and aerobic zone. Digester is used for the stabilization of sludge allowing a large fraction of the sludge organic matter to decompose under anaerobic conditions to carbon dioxide and methane.

A2O with pre-clarification is system where pre- clarification is followed by A2O system to remove organic carbon, nitrogen, and phosphorus^51^. Anaerobic digestion is used to stabilize the sludge coming from pre-clarification and final clarification units^52^.

### Establishment of decision matrices

Since four different wastewater treatment plants, which are predicted to be of critical importance for Istanbul, are A_1_, A_2_, A_3_ and A_4_ as explained above, the set of alternatives is defined as $A= \left\{{A}_{1}, {A}_{2}, {A}_{3}, {A}_{4}\right\}$. In the decision model, four main criteria related to the sustainability aspects of WWTP technologies and 24 sub-criteria were determined as shown in Fig. 1, and the set of sub criteria is $C= \left\{{C}_{1}, {C}_{2}, \dots , {C}_{24}\right\}$. The crisp data obtained from real scale WWTPs belonging to sub-criteria are as listed in Table 4. The proposed fuzzy decision making framework comprises three main phases as namely data transformation, data normalization, and calculation of dominance of each alternatives to rank. The data transformation phase performed with two different approaches depending on the qualitative and quantitative criteria. For the crisp values of quantitative data are transformed to TrFNs with respect to 20% uncertainty of condition and 5% of symmetric uncertainty around the mean calculated by ${a}_{1}=t-0.2 t, {a}_{2}= t-0.05 t, {a}_{3 =}t+0.05 t,$ and ${a}_{4=} t+0.2 t$.

**Table 4:** Crisp and linguistic data of sub-criteria for WWTP technology selection problem.

Sub-criteria	Unit	Treatment process alternatives			
		A1	A2	A3	A4
C11	KW/year for 1 m^3^ wastewater	39,069,360	21,486,488	54,109,275	48,473,924
C12	kg/year for 1 m^3^ wastewater	30,996,000	51,963,177	111,795,652	34,414,044
C13	m^3^/year	–	10,396,987	338,555	692,579
C21	USD	138,650,000	140,000,000	133,318,000	155,197,000
C22	m^2^	550,000	329,000	430,000	230,862
C23	USD/year for 1 m^3^ wastewater	6,500,000	4,700,000	8,500,000	6,000,000
C24	%	42	–	40	57
C25	USD/kg-Year for 1 m^3^ wastewater	925,540.56	1,551,620.47	3,338,218.17	1,027,692.93
C31	%	94.2	96.3	92.6	97.0
C32		80.8	93.3	85.8	97.0
C33		88.3	93.7	81.6	92.5
C34		74.7	89.0	56.8	75.0
C35		86.9	75.8	63.7	60.0
C36	1–10 scale	5	7	8	7
C37		7	8	7	8
C38		2	8	8	8
C39		2	8	8	8
C310		8	9	8	9
C311		6	10	9	10
C41		2	7	6	6
C42		7	8	7	7
C43		5	7	8	8
C44		6	8	8	8
C45		6	7	9	9

The approach to transform qualitative data consists of two separate steps. First, subjective judgements are evaluated by AHP and, in the second part evaluated data are expressed with linear fuzzy weights. After the transformation phase, both the subjective and objective data is normalized in order to create decision matrix with normalized fuzzy weights. The normalized fuzzy weighting matrix is obtained by Eq. (15). Data normalization provides a classification of data as cost and benefit to provide data compatibility. Data transformation and data normalization matrices are available on Supplementary Information.

## Results analysis

### Individual weight of criteria

**Figure 5:**
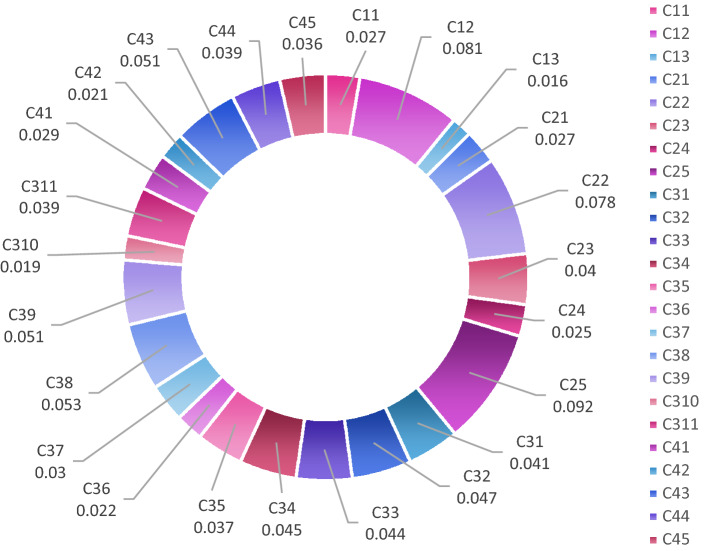
The aggregated criteria weights distributions. The aggregated weights of criteria employs to produce relative weight of each criterion provided ranking of the alternatives according to the loss-averse behavior of the DMs.

In order to obtain relative criteria, an approached weight calculation is developed on the base of α-cut set, which is shown in Fig. 3. The final weighting calculation is a crucial role in reaching compromise solution by α-cut sets which is defined by the membership function of in Eq. (17)–(20). The weighting vectors at each alpha level incorporate the methodology discussed in “Determination of criteria weights” section depending on the optimism index of the DMs, in order to enable an evaluation of the weighting of subjective judgements and objective data related to risk reverse or risk seeking behavior. This weighting procedure contributes to a decision-making process that is compatible with the TODIM method. The relative weights of the aggregated weights to be used in the TODIM method used for ranking the alternatives are calculated according to Eq. (11). The individual or relative weight of criteria calculation necessitates ascertaining reference criteria. The individual weight of criteria employs the calculation of the dominance degree of alternatives. The obtained sets of aggregated criteria are shown in Fig. 5.

Such an approach allows a clear numerical demonstration of the dominance of one alternative over another. The aggregated weight with the highest value corresponds the reference criteria used to calculate relative weights. Figure 5 clearly shows that the reference criterion (RC) had the highest aggregated weight C25 (sludge disposal cost). Another with higher aggregated weight is C12 (sludge generation). The weighting results show that sludge plays crucial role to select optimal alternative for WWTPs. In real life problem, sewage sludge is positioned as a by-product that is difficult to dispose of and high disposal costs as an insurmountable barrier. This point of view is effective in the subjective assessment of the experts and, due to the high sludge disposal costs, is also reflected in the weightings with quantitative data.

**Figure 6:**
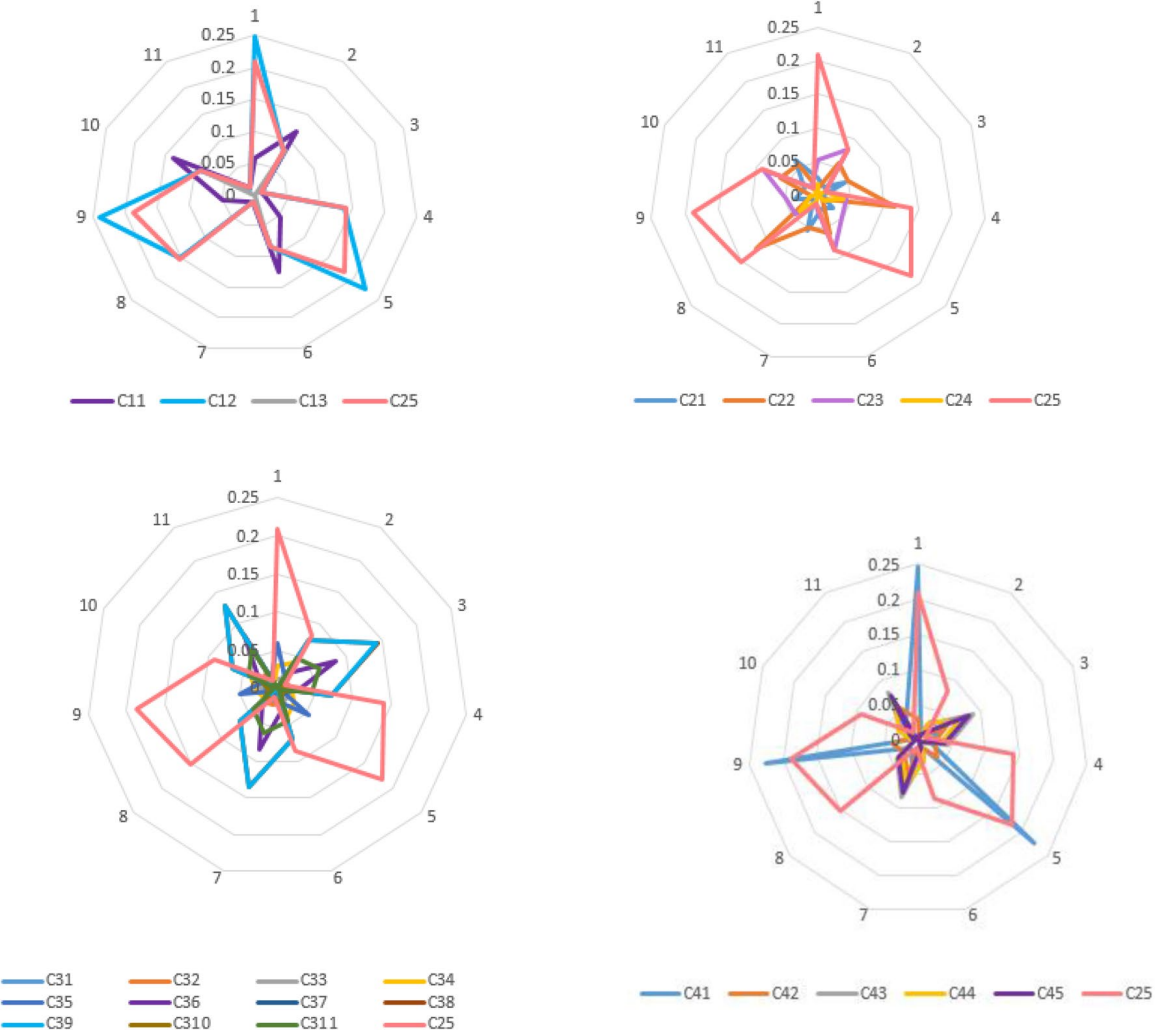
The objective weights relative to the reference criterion.

**Figure 7:**
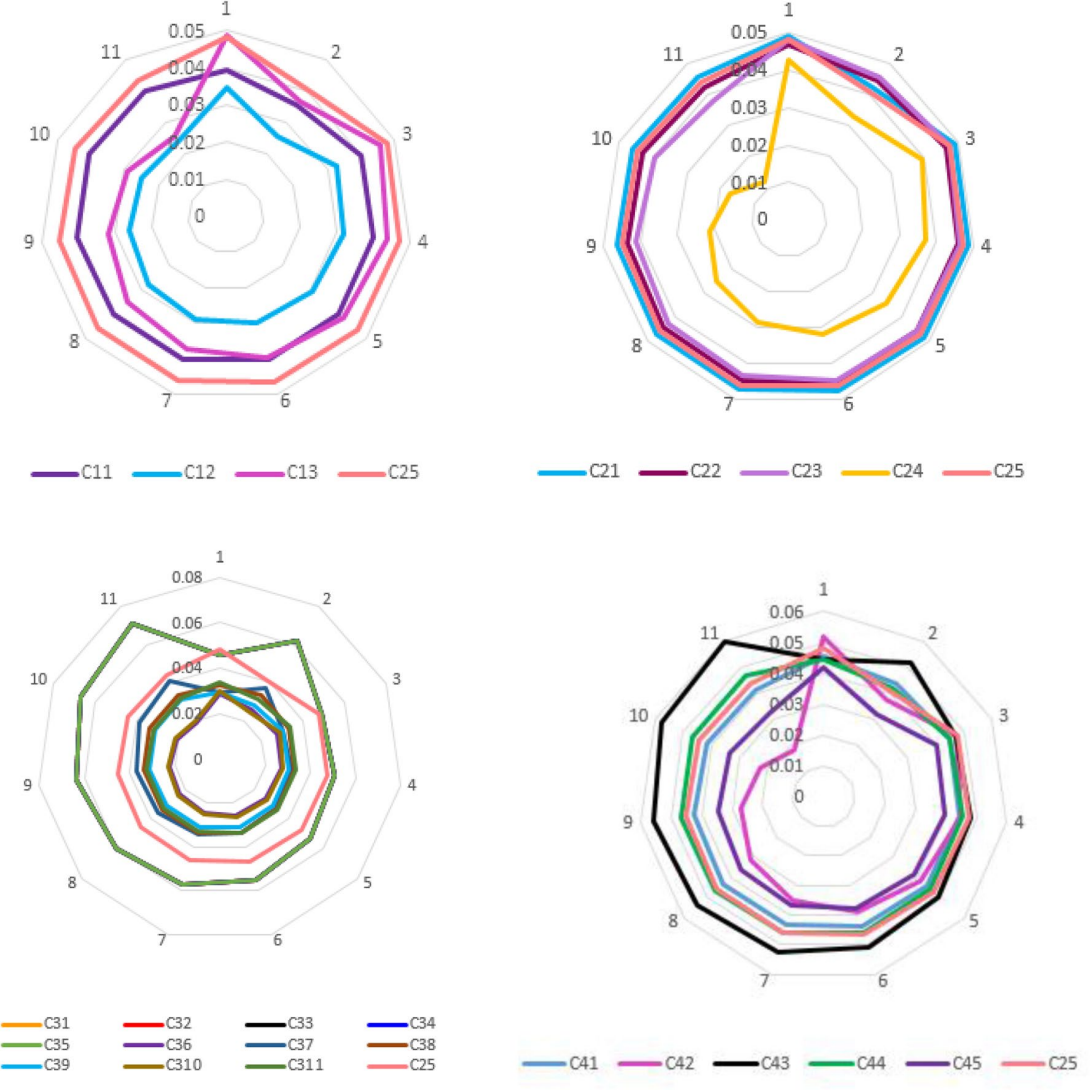
The status of each subjective weight relative to the reference criterion found.

The dominance of the objective and subjective data weights, which are the components of the aggregated weights, when selecting the reference criterion is shown in Figs. 6 and 7. The graphic interpretation of the positions of the objective and subjective data weights according to the calculated reference criterion weight clearly demonstrates the dominant weight set based on α-cuts.

### Calculation of the global dominance of each alternatives

After completion the weighting phase of the decision-making process has been completed, to evaluate the most suitable process selection of WWTP, the global dominance degree is calculated. To achieve global dominance of alternatives the procedure summarized as follows.

i. According to the evaluate gain or loss condition through Eq. (23), gain or loss is calculated based on α-cuts by Eq. (22) and where θ = 2.25.
ii. Obtaining the dominance of each alternatives from Eq. (24)
iii. Evaluating overall dominance degree of the alternatives Eq. (25)
iv. Calculating global dominance and average global dominance degree Eqs. (26) and (27), respectively.
v. Ranking of alternatives.

The dominance degree matrix of comparison between two alternatives at each alpha level sets are shown below

$${\phi }_{1}=\left[\begin{array}{cccc}0& -3.1& -4.4& -1.4\\ -11.8& 0& -10.7& -6.7\\ -4.7& 0.8& 0& 0.8\\ -10.3& -2.2& -5.8& 0\end{array}\right] { \phi }_{2}=\left[\begin{array}{cccc}0& -3.5& -4.5& -1.4\\ -12.7& 0& -11.6& -7.0\\ -7.0& 0.31& 0& 0.82\\ -11.6& -2.8& -6.2& 0\end{array}\right]$$

$${\phi }_{3}=\left[\begin{array}{cccc}0& -3.9& -4.8& -1.9\\ -13.0& 0& -12.5& -7.4\\ -8.4& 0.11& 0& 0.92\\ -12.7& -3.4& -7.3& 0\end{array}\right] {\phi }_{4}=\left[\begin{array}{cccc}0& -4.2& -5.5& -2.2\\ -16.0& 0& -13& -7.8\\ -9.6& -0.04& 0& 1.04\\ -14.4& -4.02& -8.6& 0\end{array}\right]$$

$${\phi }_{5}=\left[\begin{array}{cccc}0& -4.6& -6.7& -2.5\\ -18& 0& -14& -8.3\\ -11.0& -0.18& 0& 1.15\\ -16.9& -4.6& -9.7& 0\end{array}\right] {\phi }_{6}=\left[\begin{array}{cccc}0& -5.3& -8.4& -2.7\\ -20.5& 0& -16& -8.9\\ -12.9& -0.3& 0& 1.3\\ -19& -5.2& -11.4& 0\end{array}\right]$$

$${\phi }_{7}=\left[\begin{array}{cccc}0& -5.9& -10& -3.0\\ -23& 0& -17.8& -10\\ -15& -1.0& 0& 1.5\\ -22& -6.5& -13& 0\end{array}\right] {\phi }_{8}=\left[\begin{array}{cccc}0& -6.7& -11.5& -4.1\\ -26& 0& -20& -12\\ -17& -1.5& 0& 1.7\\ -25& -7.5& -15& 0\end{array}\right]$$

$${\phi }_{9}=\left[\begin{array}{cccc}0& -7.6& -13& -4.9\\ -30& 0& -22.5& -14\\ -20& -1.8& 0& 1.9\\ -28.5& -8.7& -17& 0\end{array}\right]{\phi }_{10}=\left[\begin{array}{cccc}0& -9.2& -15.5& -5.8\\ -36.0& 0& -27.0& -16.0\\ -23.0& -4.0& 0& 1.09\\ -33.4& -2.2& -5.8& 0\end{array}\right]$$

$${\phi }_{11}=\left[\begin{array}{cccc}0& -12.1& -18.5& -7.7\\ -44.7& 0& -33.6& -19.7\\ -27.7& -7.3& 0& 0.3\\ -41.7& -15.5& -27.4& 0\end{array}\right]$$

**Figure 8:**
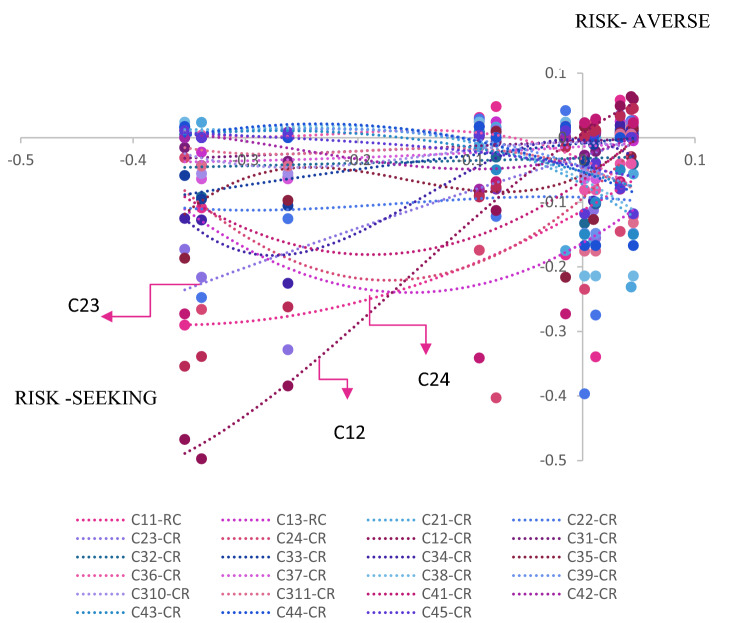
Risk-averse and risk seeking trend curves of criteria. Risk seeking and risk averse curves were obtained from the relative dominance degree of one alternative over other related to criteria to show how criteria impacts the overall dominance degree of alternatives. According to the reference criterion (C25) operational and maintenance cost (C23), energy savings (C24) and sludge generation (C12) criteria have the greatest influence on the determination of the overall dominance degree according to gain and loss value of criteria.

This overall dominance results are discussed with the graphical demonstration in Fig. 8 in terms of gain and loss calculation via Eq. (24).

The overall dominance degree $\delta ({A}_{i})$ of alternative ${A}_{i}$ is calculated by using Eq. (25) and $\delta ({A}_{i})$ are obtained as follows:

$${\delta }_{1}({A}_{1})=-8.9; {\delta }_{1}({A}_{2})=-29.2; {\delta }_{1}({A}_{3})=-3.1; {\delta }_{1}({A}_{4})=- 18.3$$

$${\delta }_{2}({A}_{1})=-9.4; {\delta }_{2}({A}_{2})=-31.3; {\delta }_{2}({A}_{3})=-5.87; {\delta }_{2}({A}_{4})=- 20.6$$

$${\delta }_{3}({A}_{1})=-10.6; {\delta }_{3}({A}_{2})=-32.9; {\delta }_{3}({A}_{3})=-7.37; {\delta }_{3}({A}_{4})=-23.4$$

$${\delta }_{4}({A}_{1})=-11.9; {\delta }_{4}({A}_{2})=-36.8; {\delta }_{4}({A}_{3})=-8.6; {\delta }_{4}({A}_{4})=-27$$

$${\delta }_{5}({A}_{1})=-13.8; {\delta }_{5}({A}_{2})=-40.3; {\delta }_{5}({A}_{3})=-10.3; {\delta }_{5}({A}_{4})=-31.2$$

$${\delta }_{6}({A}_{1})=-16.4; {\delta }_{6}({A}_{2})=-45.5; {\delta }_{6}({A}_{3})=-11.9; {\delta }_{6}({A}_{4})=-35.6$$

$${\delta }_{7}({A}_{1})=-18.9; {\delta }_{7}({A}_{2})=-40.3; {\delta }_{7}({A}_{3})=-13.5; {\delta }_{7}({A}_{4})=-31.2$$

$${\delta }_{8}({A}_{1})=-22.3; {\delta }_{8}({A}_{2})=-58.0; {\delta }_{8}({A}_{3})=-16.8; {\delta }_{8}({A}_{4})=-47.5$$

$${\delta }_{9}({A}_{1})=-25.5; {\delta }_{9}({A}_{2})=-66.5; {\delta }_{9}({A}_{3})=-19.9; {\delta }_{9}({A}_{4})=-54.2$$

$${\delta }_{10}({A}_{1})=-30.5; {\delta }_{10}({A}_{2})=-79.0; {\delta }_{10}({A}_{3})=-25.9; {\delta }_{10}({A}_{4})=-41.4$$

$${\delta }_{11}({A}_{1})=-38.3; {\delta }_{11}({A}_{2})=-9.8; {\delta }_{11}({A}_{3})=-34.7; {\delta }_{11}({A}_{4})=-84.6$$

The global dominance of the alternatives is determined according to Eq. (26) considering ${\delta }_{\alpha }\left({A}_{i}, {A}_{j}\right)$ function and by aligning the global dominance according to ${\delta }_{\alpha }\left({A}_{i}, {A}_{j}\right)$ function. It facilitated ranking of alternatives with respect to overall dominance degrees that represented in Table 5.

**Table 5:** The global dominance of alternatives for each α-cuts and rank of the alternatives for θ = 2.25.

α											
${\xi }_{\alpha }\left({A}_{i}\right)$	0	0.1	0.2	0.3	0.4	0.5	0.6	0.7	0.8	0.9	1.0
A1	0	0	0	0	0	0	0	0	0	0	0
A2	1	1	1	1	1	0.989	0.971	0.977	0.98	0.95	0.911
A3	0.242	0.33	0.346	0.373	0.395	0.378	0.384	0.391	0.4	0.41	0.383
A4	0.868	0.93	0.95	0.973	0.993	1	1	1	1	1	1
Rank	2-4-3-1	2-4-3-1	2-4-3-1	2-4-3-1	2-4-3-1	4-2-3-1	4-2-3-1	4-2-3-1	4-2-3-1	4-2-3-1	4-2-3-1

The expression ${\delta }_{\alpha }\left({\stackrel{\sim }{\mathrm{A}}}_{\mathrm{i}}, \tilde{A}_{\mathrm{j}}\right)$ indicates the performance of each alternative based on each sub-criteria and the superiority of A_i_ over A_j_ for each α-cuts.

The global values ${\xi }_{\alpha }\left({A}_{i}\right)$ allows a clear ordering for appropriate selection. To compare alternative i with alternative j, the function ${\delta }_{\alpha }\left({A}_{i}, {A}_{j}\right)$ can be used, which is a value function and is expressed by computing ${\phi }_{k}^{\alpha }\left({A}_{i}, {A}_{j}\right)$ will. The determination of global dominance is carried out in two ways in order to obtain a compromise solution. First, the global degree is calculated using α-cut sets, the ranking options for selecting suitable WWTPs depending on the DM perspective. The second is to calculate the average global dominance to demonstrate the possible optimal set of solutions. The results show that the ranking of the alternatives based on the average global dominance degree for the selection of the WWTP process is: A_2_ > A_4_ > A_3_ > A_1_. The average global dominance degree is calculated as $\overline{\xi } \left({A}_{i}\right)=\left\{\mathrm{0,0.979}, 0.384, 0.974\right\}$.

In order to deal with the uncertainty that arises from subjective judgments and incomplete information that may be contained in the objective data, an effort id made to maintain the uncertain conditions until the end of the decision-making process by using the data presented with TrFNs were fuzzified and, alpha cut series without defuzzification. The model offers decision makers the opportunity to choose according to risk-taking or risk-avoidance behavior. The average global dominance degree of alternatives ranks the alternatives depends on the attenuation factor $\left(\theta \right),$ which reflects the DM’s point of view regarding risk aversion or risk seeking behavior.

## Discussion

In real life scenarios, dynamic environmental conditions lead to urgent decisions that may affect the psychological base behind behavior of DMs^53^. Thankfully, limitations such as governmental regulations, limited investment cost, compelling features of energy recovery, or sludge disposal can facilitate standardization the perception of risk in technology selection for wastewater treatment plants^54^. Determining the criteria that affect the perception of risk can ensure that the decisions are more rational and compatible with real cases. From this vantage point, the current study proposes a systematic approach that reflecting the decision making mechanism of risk aversion and revealing criteria set that manipulate the risk perception of DMs. By calculating the dominance degree of the alternatives, it is shown that the model enables options to rank alternatives in terms of risk-seekers or risk aversion behavior of DMs. The results showed that the decision maker's attitude towards risk aversion or risk-seeking the model predicts the cost of sludge disposal (C25) as the reference criterion. When calculating the dominance degree of the alternatives, it is shown that the model eliminates the choices in the risk-seeking tendency and highlights the gain values in the risk aversion tendency as shown in Fig. 8. The model performs by determining the dominance degrees depending on gain values of the criteria that fit into the sigmoidal function (S-shaped) for choices that tend to risk-averse, while reducing the effectiveness of the loss values that tend to risk-seeking. As a result, the criteria that have a high weight in the selections with a tendency of risk aversion come to the fore and determine the dominance degree of alternatives. The results implies that alternative A_2_ (A2O without pre-clarification) is the optimal technology of wastewater treatment plants in terms of sustainability. According to the current decision process based on the sustainability perspective, ‘’A2O without pre-clarification’’ has emerged as the most ideal and sustainable process according to risk aversion approach. Sludge disposal cost (C25), which is the most effective criterion in this decision process, also plays a key role in real-life risk-oriented technology choices for wastewater treatment.

Sludge, which is recognized as an environmental problem due to the difficulties in disposal for WWTPs, is one of the main factor affecting the energy savings amounts, operational and maintenance problems and operating costs in the facilities^55^. Taking everything into consideration, the proposed decision process reveals a realistic ranking for technology selection of WWTPs rather than ideal one. Although, in real life, the investment costs are considered more in the design or operation of the WWTPs in the risk-avoidance choices of the decision makers, it emerges in a systematic decision making approach supported by scientific methods that the determining criteria should be environmental factors such as sludge for the implementation of correct sustainability policies.

### Sensitivity and comparative analysis

The sensitivity analysis was performed by changing the value of θ in order to obtaining comparative results regarding the risk aversion or risk seeking trend of DMs^56^. Table 6 shows the variations in the results along with θ values and average global dominance degree of alternatives and, ranking.

**Table 6:** The sensitivity analysis changing θ value.

θ	${\xi }_{i}$				Ranking of alternatives
	A_1_	A_2_	A_3_	A_4_	
0.1	0	0.982	0.416	0.973	A_2_ > A_4_ > A_3_ > A_1_
1	0	0.981	0.402	0.974	A_2_ > A_4_ > A_3_ > A_1_
2	0	0.980	0.388	0.975	A_2_ > A_4_ > A_3_ > A_1_
2.25	0	0.979	0.384	0.975	A_2_ > A_4_ > A_3_ > A_1_
3	0	0.978	0.374	0.976	A_2_ > A_4_ > A_3_ > A_1_
4	0	0.977	0.360	0.976	A_2_ > A_4_ > A_3_ > A_1_
5	0	0.976	0.347	0.977	A_4_ > A_2_ > A_3_ > A_1_
10	0	0.970	0.285	0.979	A_4_ > A_2_ > A_3_ > A_1_

The sensitivity analysis results in Table 6 show that the difference between the global dominance degree of competing alternatives A_2_ and A_4_ is increase with respect to smaller risk aversion perception. The value of θ shows that the different psychological behavior in terms of risk aversion. The smaller θ expresses that a more risk averse behavior manages the ranking of alternatives. For example, θ = 0.1 means that a higher risk aversion is indicated and θ = 10 the experts prepare to taking risks so that the risk-seeking behavior manipulates the decisions. For this reason, the proposed decision-making model leads to consistent results according to the sensitivity analysis. While the compromise solution for a decision maker with a risk-aversion perspective is *2* > *4* > *3* > *1* for the optimal process selection of wastewater treatment plant, the result for a decision-maker with a risk-seeking perspective changes as *4* > *2* > *3* > *1*.

For the comparative study, TOPSIS, fuzzy TOPSIS and, intuitionistic TOPSIS and, intuitionistic VIKOR were used. Fuzzy TOPSIS under TrFN was applied for the comparative analysis^39^. Fuzzy TOPSIS is a methodology to rank of alternatives based on calculation of shortest distance from the positive ideal solution and the farthest distance from the negative ideal solution^57^. After the normalization process of the qualitative and quantitative data, the weights of which are calculated by expressing with TrFNs, the fuzzy TOPSIS method leads to the calculation of the positive fuzzy ideal solution and the fuzzy negative ideal solution. The distance of each alternative from the fuzzy positive ideal solution and the fuzzy negative ideal solution is calculated, and the alternatives are ranked according to their closeness coefficients values. A detailed procedure of fuzzy TOPSIS under the TrFN can be referred to^58^. In addition, in order to demonstrate the sensitivity of the proposed extended fuzzy TODIM methodology, the intuitionistic VIKOR (IF-VIKOR) and the intuitionistic TOPSIS (IF-TOPSIS) method are applied to the procedure introduced by Alkafaas et al. in 2020^59^ and Uyanik et al. in 2020^60^. The comparison of ranking results are represented in Table 7. The main factor of comparative analysis revealed reasonable output due to both of methods based on reference dependent approach and distance calculation.

**Table 7:** The sensitivity analysis performed by TOPSIS, fuzzy TOPSIS, IF TOPSIS and, IF VIKOR.

	TOPSIS	F-TOPSIS	IF-TOPSIS	IF-VIKOR
	CC_i_	CC_i_	C_i_	Q
A_1_	0.528	0.530	0.9208	0.9972
A_2_	0.659	0.584	0.9318	0.0018
A_3_	0.336	0.223	0.9280	0.7432
A_4_	0.544	0.558	0.9367	0.5000
Rank	A_2_ > A_4_ > A_1_ > A_3_	A_2_ > A_4_ > A_1_ > A_3_	A_4_ > A_2_ > A_3_ > A_1_	A_2_ > A_4_ > A_3_ > A_1_

Proposed model in this study reflects much more clear the dominance of the alternatives with respect to psychological behavior over each other comparing with TOPSIS and fuzzy TOPSIS. In the comparative study performed with the IF-TOPSIS and IF-VIKOR methods, IF-TOPSIS revealed the same ranking of risk-seeking behavior in the ranking of the competing alternatives A_2_ and A_4_ compared to the proposed method, while IF-VIKOR produced a similar ranking with less risk-aversion psychological behavior.

## Conclusion

In this proposed decision-making framework, the technology selection for wastewater treatment was evaluated using a holistic approach considering the environmental, economic, technical and social aspects of sustainability. According to the risk aversion approach, the economic criteria have the most weight in the model, ‘’the sludge disposal cost’’, which is a sub-criterion of economic factors, has become the decisive criterion to compare the relative dominance of alternatives. Heterogeneous data including both qualitative and quantitative data, were normalized in the fuzzy environment for use in the proposed decision model. The best compromise solutions in terms of alpha cut series for wastewater treatment technology selection have been achieved based on a risk-seeking and risk aversion approach of the DMs. The performance indicators were assessed to support the decision-making process operated with the TODIM methodology in a fuzzy environment, which seems to offer the most appropriate decision-making strategy based on behavioral characteristics of DMs and considering emergencies to effectively control the decision-making. Using a methodology based on fuzzy and risk oriented decision-making provided an opportunity to include not only objective data, such as inaccurate or insufficient technical information, but also subjective judgements of DMs in terms of personal experience or level of knowledge and awareness in the decision-making process. As future work, the proposed decision-making framework can be evaluated under the intuitionistic fuzzy environment to express the uncertainty of decision information derived from subjective judgements and improved to rank alternatives, and hybrid models can be performed to compare water/wastewater treatment processes.

## Supplementary Information

Supplementary Table 1.

Supplementary Table 2.

Supplementary Table 3.

Supplementary Table 4.

Supplementary Table 5.

Supplementary Table 6.

Supplementary Table 7.
