# Supplementary material for: A novel fuzzy framework for technology selection of sustainable wastewater treatment plants based on TODIM methodology in developing urban areas
Source: Sci Rep. 2022 May 25;12:8800. doi: 10.1038/s41598-022-12643-1 (PMC9132933; doi:10.1038/s41598-022-12643-1)
Supplement: Supplementary file 1 — Supplementary Table 1. [file 41598_2022_12643_MOESM1_ESM.docx]

**Supplementary Table 1.** Normalized weights of subjective judgements calculated according to Eq.(15)

| *Criteria* |  | | | |
| --- | --- | --- | --- | --- |
|  | *b_1_* | *b_2_* | *b_3_* | *b_4_* |
| C11 | 0.74 | 0.13 | 0.33 | 0.63 |
| C12 | 0 | 0.52 | 0.33 | 0.30 |
| C13 | 0.72 | 0.56 | 0.59 | 1 |
| C21 | 0.67 | 0.57 | 1.00 | 0.89 |
| C22 | 0.65 | 0.46 | 0.96 | 0.74 |
| C23 | 0.72 | 0.93 | 0.59 | 0.74 |
| C24 | 0.38 | 0.34 | 0.78 | 0.41 |
| C25 | 0.85 | 0.96 | 0.63 | 0.54 |
| C31 | 0.38 | 0.93 | 0.87 | 0.83 |
| C32 | 0.38 | 0.93 | 0.87 | 0.83 |
| C33 | 0.38 | 0.93 | 0.87 | 0.83 |
| C34 | 0.38 | 0.93 | 0.87 | 0.83 |
| C35 | 0.38 | 0.93 | 0.87 | 0.83 |
| C36 | 0.09 | 0.03 | 0.04 | 0 |
| C37 | 0.13 | 0.09 | 0.10 | 0 |
| C38 | 0.22 | 0.31 | 0.19 | 0.6 |
| C39 | 0.13 | 0.16 | 0.06 | 0 |
| C310 | 0.09 | 0.19 | 0.03 | 0 |
| C311 | 0.25 | 0.28 | 0.19 | 0.22 |
| C41 | 0.61 | 0.89 | 0.67 | 0.41 |
| C42 | 0.85 | 0.80 | 0.81 | 0.98 |
| C43 | 0.5 | 0.75 | 0.72 | 0.66 |
| C44 | 0.59 | 0.53 | 0.69 | 0.66 |
| C45 | 0.59 | 0.75 | 0.72 | 0.66 |
