# Supplementary material for: A novel fuzzy framework for technology selection of sustainable wastewater treatment plants based on TODIM methodology in developing urban areas
Source: Sci Rep. 2022 May 25;12:8800. doi: 10.1038/s41598-022-12643-1 (PMC9132933; doi:10.1038/s41598-022-12643-1)
Supplement: Supplementary file 2 — Supplementary Table 2. [file 41598_2022_12643_MOESM2_ESM.docx]

**Supplementary Table 2**. Normalized weights matrix of evaluated subjective judgements calculated through Eq. (17) and Eq.(18).

| α-cuts | | | | | | | | | | | | | |
| --- | --- | --- | --- | --- | --- | --- | --- | --- | --- | --- | --- | --- | --- |
|  | *0* | *0.1* | | *0.2* | ***0.3*** | | *0.4* | *0.5* | *0.6* | *0.7* | *0.8* | *0.9* | *1.0* |
| Criteria |  |  |  | | |  |  |  |  |  |  |  |  |
| C11 | 0.0395 | 0.0356 | 0.0398 | | | 0.0399 | 0.0400 | 0.0401 | 0.0402 | 0.0403 | 0.0403 | 0.0403 | 0.0402 |
| C12 | 0.0347 | 0.0255 | 0.0329 | | | 0.0320 | 0.0310 | 0.0300 | 0.0289 | 0.0278 | 0.0265 | 0.0252 | 0.0238 |
| C13 | 0.0490 | 0.0370 | 0.0457 | | | 0.0439 | 0.0419 | 0.0397 | 0.0374 | 0.0349 | 0.0321 | 0.0291 | 0.0258 |
| C21 | 0.0492 | 0.0419 | 0.0487 | | | 0.0484 | 0.0482 | 0.0478 | 0.0475 | 0.0470 | 0.0465 | 0.0460 | 0.0453 |
| C22 | 0.0471 | 0.0440 | 0.0465 | | | 0.0461 | 0.0457 | 0.0453 | 0.0447 | 0.0442 | 0.0435 | 0.0427 | 0.0419 |
| C23 | 0.0485 | 0.0452 | 0.0472 | | | 0.0465 | 0.0457 | 0.0447 | 0.0437 | 0.0425 | 0.0412 | 0.0396 | 0.0378 |
| C24 | 0.0430 | 0.0326 | 0.0391 | | | 0.0370 | 0.0345 | 0.0318 | 0.0288 | 0.0253 | 0.0215 | 0.0171 | 0.0120 |
| C25 | 0.0482 | 0.0406 | 0.0476 | | | 0.0473 | 0.0470 | 0.0466 | 0.0461 | 0.0456 | 0.0451 | 0.0445 | 0.0437 |
| C31 | 0.0456 | 0.0622 | 0.0488 | | | 0.0507 | 0.0527 | 0.0549 | 0.0574 | 0.0602 | 0.0633 | 0.0669 | 0.0709 |
| C32 | 0.0456 | 0.0622 | 0.0488 | | | 0.0507 | 0.0527 | 0.0549 | 0.0574 | 0.0602 | 0.0633 | 0.0669 | 0.0709 |
| C33 | 0.0456 | 0.0622 | 0.048 | | | 0.0507 | 0.0527 | 0.0549 | 0.0574 | 0.0602 | 0.0633 | 0.0669 | 0.0709 |
| C34 | 0.0456 | 0.0622 | 0.0488 | | | 0.0507 | 0.0527 | 0.0549 | 0.0574 | 0.0602 | 0.0633 | 0.0669 | 0.0709 |
| C35 | 0.0456 | 0.0622 | 0.0488 | | | 0.0507 | 0.0527 | 0.0549 | 0.0574 | 0.0602 | 0.0633 | 0.0669 | 0.0709 |
| C36 | 0.0288 | 0.0260 | 0.0276 | | | 0.0270 | 0.0262 | 0.0254 | 0.0244 | 0.0233 | 0.0221 | 0.0207 | 0.0190 |
| C37 | 0.0293 | 0.0371 | 0.0307 | | | 0.0315 | 0.0324 | 0.0334 | 0.0345 | 0.0358 | 0.0373 | 0.0389 | 0.0409 |
| C38 | 0.0328 | 0.0336 | 0.0330 | | | 0.0331 | 0.0332 | 0.0333 | 0.0334 | 0.0335 | 0.0336 | 0.0338 | 0.0339 |
| C39 | 0.0297 | 0.0280 | 0.0301 | | | 0.0303 | 0.0305 | 0.0307 | 0.0309 | 0.0311 | 0.0313 | 0.0314 | 0.0315 |
| C310 | 0.0302 | 0.0242 | 0.0289 | | | 0.0280 | 0.0272 | 0.0263 | 0.0253 | 0.0242 | 0.0230 | 0.0217 | 0.0203 |
| C311 | 0.0340 | 0.0309 | 0.0338 | | | 0.0336 | 0.0335 | 0.0333 | 0.0331 | 0.0329 | 0.0326 | 0.0323 | 0.0319 |
| C41 | 0.0454 | 0.0436 | 0.0449 | | | 0.0446 | 0.0443 | 0.0439 | 0.0435 | 0.0430 | 0.0424 | 0.0418 | 0.0410 |
| C42 | 0.0520 | 0.0375 | 0.0473 | | | 0.0447 | 0.0418 | 0.0387 | 0.0352 | 0.0315 | 0.0273 | 0.0228 | 0.0177 |
| C43 | 0.0448 | 0.0518 | 0.0470 | | | 0.0482 | 0.0495 | 0.0509 | 0.0524 | 0.0540 | 0.0557 | 0.0575 | 0.0595 |
| C44 | 0.0443 | 0.0417 | 0.0449 | | | 0.0452 | 0.0455 | 0.0458 | 0.0461 | 0.0463 | 0.0466 | 0.0468 | 0.0469 |
| C45 | 0.0417 | 0.0320 | 0.0398 | | | 0.0399 | 0.0386 | 0.0377 | 0.0368 | 0.0358 | 0.0347 | 0.0335 | 0.0323 |
