# Supplementary material for: A novel fuzzy framework for technology selection of sustainable wastewater treatment plants based on TODIM methodology in developing urban areas
Source: Sci Rep. 2022 May 25;12:8800. doi: 10.1038/s41598-022-12643-1 (PMC9132933; doi:10.1038/s41598-022-12643-1)
Supplement: Supplementary file 3 — Supplementary Table 3. [file 41598_2022_12643_MOESM3_ESM.docx]

**Supplementary Table 3.** Data transformation matrix of alternatives A1 and A2

| Criteria | *A1* | | | | *A2* | | | |
| --- | --- | --- | --- | --- | --- | --- | --- | --- |
|  | *a_1_* | *a_2_* | *a_3_* | *a_4_* | *a_1_* | *a_2_* | *a_3_* | *a_4_* |
| C11 | 31,255,488 | 37,115,892 | 41,022,828 | 46,883,232 | 17,189,190.40 | 20,412,163.60 | 22,560,812.40 | 25,783,785.60 |
| C12 | 24,796,800 | 29,446,200 | 32,545,800 | 37,195,200 | 41,570,541.60 | 49,365,018.15 | 54,561,335.85 | 62,355,812.40 |
| C13 | 0 | 0 | 0 | 0 | 8,317,589.60 | 9,877,137.65 | 10,916,836.35 | 12,476,384.40 |
| C21 | 110,255,488 | 131,717,500 | 145,582,500 | 166,380,000 | 124,157,600 | 147,437.,150 | 162,956,850 | 186,236,400 |
| C22 | 440,000 | 525,500 | 577,500 | 660,000 | 263,200 | 522,500 | 577,500 | 660,000 |
| C23 | 5,200,000 | 6,175,000 | 6,825,000 | 7,800,000 | 3,760,000 | 4,465,000 | 4,935,000 | 5,640,000 |
| C24 | 33.6 | 39.90 | 44.10 | 50.40 | 0 | 0 | 0 | 0 |
| C25 | 740,432.45 | 879,263.53 | 971,817.59 | 1,110,648.67 | 1,241,296.37 | 1,474,039.44 | 11,629,201.49 | 1,861,944.56 |
| C31 | 75.33 | 89.46 | 98.88 | 113 | 77.07 | 91.52 | 101.15 | 115.60 |
| C32 | 64.67 | 76.79 | 84.88 | 97 | 74.67 | 88.67 | 98 | 112 |
| C33 | 70.67 | 83.92 | 92.75 | 106 | 74.93 | 88.98 | 98.35 | 112.40 |
| C34 | 59.73 | 70.93 | 78.40 | 89.60 | 71.20 | 84.55 | 93.45 | 106.80 |
| C35 | 69.52 | 82.56 | 91.25 | 104.28 | 60,61 | 71,98 | 79,56 | 90,92 |
| C36 | 4 | 4.75 | 5.25 | 6.00 | 5.60 | 6.65 | 7.35 | 8.40 |
| C37 | 5.60 | 6.65 | 7.35 | 8.40 | 6.40 | 7.60 | 8.40 | 9.60 |
| C38 | 1.60 | 1.90 | 2,10 | 2,40 | 6,40 | 7,60 | 8,40 | 9,60 |
| C39 | 1,60 | 1,90 | 2,10 | 2.40 | 6.40 | 7.60 | 8.40 | 9.60 |
| C310 | 6.40 | 7.60 | 8.40 | 9.60 | 7.20 | 8.55 | 9.45 | 10.80 |
| C311 | 4.80 | 5.70 | 6.30 | 7.20 | 8 | 9.50 | 10.50 | 12 |
| C41 | 1.60 | 1.90 | 2.10 | 2.40 | 5.60 | 6.65 | 7.35 | 8.40 |
| C42 | 5.60 | 6.65 | 7.35 | 8.40 | 6.40 | 7.60 | 8.40 | 9.60 |
| C43 | 4 | 4.75 | 5.25 | 6.00 | 5.60 | 6.65 | 7.35 | 8.40 |
| C44 | 4.80 | 5.70 | 6.30 | 7.20 | 6.40 | 7.60 | 8.40 | 9.60 |
| C45 | 4.80 | 5.70 | 6.30 | 7.20 | 5.60 | 6.65 | 7.35 | 8.40 |
