# Supplementary material for: A novel fuzzy framework for technology selection of sustainable wastewater treatment plants based on TODIM methodology in developing urban areas
Source: Sci Rep. 2022 May 25;12:8800. doi: 10.1038/s41598-022-12643-1 (PMC9132933; doi:10.1038/s41598-022-12643-1)
Supplement: Supplementary file 4 — Supplementary Table 4. [file 41598_2022_12643_MOESM4_ESM.docx]

**Supplementary Table 4.** Data transformation matrix of alternatives A3 and A4.

| Criteria | *A3* | | | | A*4* | | | |
| --- | --- | --- | --- | --- | --- | --- | --- | --- |
|  | *a_1_* | *a_2_* | *a_3_* | *a_4_* | *a_1_* | *a_2_* | *a_3_* | *a_4_* |
| C11 | 43,287,420 | 51,403,811.25 | 56,814,738.75 | 64,931,130 | 38,779,139.20 | 46,050,227.80 | 50,897,620.20 | 58,168,708.80 |
| C12 | 89,436,521.60 | 106,205,869.40 | 117,385,434.60 | 134,154,782.40 | 27,533,635.20 | 32,696,191.80 | 36,137,896.20 | 41,300,452.80 |
| C13 | 270,844 | 321,627.25 | 355,482.75 | 406,266 | 554,063.20 | 657,950.05 | 727,207.95 | 831,094.80 |
| C21 | 106,654,400 | 126,652,100 | 139,983,900 | 159,981,600 | 124,157,600 | 147,437,150 | 162,956,850 | 186,236,400 |
| C22 | 344,000 | 408,500 | 451,500 | 516,000 | 184,689.60 | 219,318.90 | 242,405.10 | 277,034.40 |
| C23 | 6,800,000 | 8,075,000 | 8,925,000 | 10,200,000 | 4,800,000 | 5,700,000 | 6,300,000 | 7,200,000 |
| C24 | 32 | 38 | 42 | 48 | 45,60 | 54,15 | 59,85 | 68,40 |
| C25 | 2,670,574.53 | 3,171,307.26 | 3,505,129.08 | 4,005,861.80 | 822,154.35 | 976,308.29 | 1,079,077.58 | 1,233,231.52 |
| C31 | 74.08 | 87.97 | 97.23 | 111.12 | 77.60 | 92.15 | 101.85 | 116.40 |
| C32 | 68.67 | 81.54 | 90.13 | 103 | 77.60 | 92.15 | 101.85 | 116.40 |
| C33 | 65.31 | 77.55 | 85.72 | 97.96 | 74; 87 | 88 | 97.13 | 111.00 |
| C34 | 45.47 | 53.99 | 59.68 | 68.20 | 60.00 | 71.25 | 78.75 | 90.00 |
| C35 | 50.93 | 60.48 | 66.85 | 76.40 | 65.07 | 77.27 | 85.40 | 97.60 |
| C36 | 6.40 | 7.60 | 8.40 | 9.60 | 5.60 | 6.65 | 7.35 | 8.40 |
| C37 | 5.60 | 6.65 | 7.35 | 8.40 | 6.40 | 7.60 | 8.40 | 9.60 |
| C38 | 6.40 | 7.60 | 8.40 | 9.60 | 6.40 | 7.60 | 8.40 | 9.60 |
| C39 | 6.40 | 7.60 | 8.40 | 9.60 | 6.40 | 7.60 | 8.40 | 9.60 |
| C310 | 6.40 | 7.60 | 8.40 | 9.60 | 7.20 | 8.55 | 9.45 | 10.80 |
| C311 | 7.20 | 8.55 | 9.45 | 10.80 | 8 | 9.50 | 10.50 | 12 |
| C41 | 4.80 | 5.70 | 6.30 | 7.20 | 4.80 | 5.70 | 6.30 | 7.20 |
| C42 | 5.60 | 6.65 | 7.35 | 8.40 | 5.60 | 6.65 | 7.35 | 8.40 |
| C43 | 6.40 | 7.60 | 8.40 | 9.60 | 6.40 | 7.60 | 8.40 | 9.60 |
| C44 | 6.40 | 7.60 | 8.40 | 9.60 | 6.40 | 7.60 | 8.40 | 9.60 |
| C45 | 7.20 | 8.55 | 9.45 | 10.80 | 7.20 | 8.55 | 9.45 | 10.80 |
