# Supplementary material for: A novel fuzzy framework for technology selection of sustainable wastewater treatment plants based on TODIM methodology in developing urban areas
Source: Sci Rep. 2022 May 25;12:8800. doi: 10.1038/s41598-022-12643-1 (PMC9132933; doi:10.1038/s41598-022-12643-1)
Supplement: Supplementary file 5 — Supplementary Table 5. [file 41598_2022_12643_MOESM5_ESM.docx]

**Supplementary Table 5.** Data normalization matrix

| Criteria | *A1* | | | | *A2* | | | | *A3* | | | | *A4A4* | | | |
| --- | --- | --- | --- | --- | --- | --- | --- | --- | --- | --- | --- | --- | --- | --- | --- | --- |
|  | *a_1_* | *a_2_* | *a_3_* | *a_4_* | *a_1_* | *a_2_* | *a_3_* | *a_4_* | *a_1_* | *a_2_* | *a_3_* | *a_4_* | *a_1_* | *a_2_* | *a_3_* | *a_4_* |
| C11 | 0.71 | 0.58 | 0.50 | 0.38 | 1 | 0.93 | 0.89 | 0.82 | 0.45 | 0.28 | 0.17 | 0 | 0.45 | 0.28 | 0.17 | 0 |
| C12 | 1 | 0.96 | 0.93 | 0.89 | 0.85 | 0.78 | 0.73 | 0.66 | 0.41 | 0.26 | 0.15 | 0 | 0.66 | 0.53 | 0.44 | 0.30 |
| C13 | 0 | 0 | 0 | 0 | 0.66 | 0.79 | 0.87 | 1 | 0 | 0.0041 | 0.0069 | 0.01 | 0.023 | 0.032 | 0.037 | 0.046 |
| C21 | 0.95 | 0.69 | 0.51 | 0.25 | 0.78 | 0.49 | 0.29 | 0 | 1 | 0.75 | 0.58 | 0.33 | 0.78 | 0.49 | 0.29 | 0 |
| C22 | 0.46 | 0.29 | 0.17 | 0 | 0.78 | 0.49 | 0.29 | 0 | 0.66 | 0.53 | 0.44 | 0.30 | 1 | 0.93 | 0.88 | 0.81 |
| C23 | 0.78 | 0.63 | 0.52 | 0.37 | 0.83 | 0.29 | 0.17 | 0 | 0.53 | 0.33 | 0.20 | 0 | 0.84 | 0.70 | 0.61 | 0.47 |
| C24 | 0.04 | 0.22 | 0.33 | 0.51 | 0 | 0 | 0 | 0 | 0 | 0,16 | 0,27 | 0,44 | 0.37 | 0.61 | 0.77 | 1 |
| C25 | 1 | 0.96 | 0.93 | 0.89 | 0.85 | 0.78 | 0.73 | 0.66 | 0.41 | 0.26 | 0.15 | 0 | 0.97 | 0.93 | 0.90 | 0.85 |
| C31 | 0.03 | 0.36 | 0.59 | 0.92 | 0.07 | 0.41 | 0.64 | 0.98 | 0 | 0.33 | 0.55 | 0.88 | 0.08 | 0.43 | 0.66 | 1 |
| C32 | 0 | 0.23 | 0.39 | 0.63 | 0.19 | 0.46 | 0.64 | 0.91 | 0.08 | 0.33 | 0.49 | 0.74 | 0.25 | 0.53 | 0.72 | 1 |
| C33 | 0.11 | 0.40 | 0.58 | 0.86 | 0.20 | 0.50 | 0.70 | 1 | 0 | 0.26 | 0.43 | 0.69 | 0.18 | 0.48 | 0.68 | 0.97 |
| C34 | 0.23 | 0.42 | 0.54 | 0.72 | 0.42 | 0.64 | 0.78 | 1 | 0 | 0.14 | 0.23 | 0.37 | 0.24 | 0.42 | 0.54 | 0.73 |
| C35 | 0.35 | 0.59 | 0.76 | 1 | 0.18 | 0.39 | 0.54 | 0.75 | 0 | 0.18 | 0.30 | 0.48 | 0 | 0.16 | 0.27 | 0.43 |
| C36 | 0 | 0.13 | 0.22 | 0.36 | 0.29 | 0.47 | 0.60 | 0.79 | 0.43 | 0.64 | 0.79 | 1 | 0.29 | 0.47 | 0.60 | 0.79 |
| C37 | 0 | 0.26 | 0.44 | 0.70 | 0.20 | 0.50 | 0.70 | 1 | 0 | 0.26 | 0.44 | 0.70 | 0.20 | 0.50 | 0.70 | 1 |
| C38 | 0 | 0.04 | 0.06 | 0.1 | 0.60 | 0.75 | 0.85 | 1 | 0.60 | 0.75 | 0.85 | 1 | 0.60 | 0.75 | 0.85 | 1 |
| C39 | 0 | 0.04 | 0.06 | 0.1 | 0.60 | 0.75 | 0.85 | 1 | 0.60 | 0.75 | 0.85 | 1 | 0.60 | 0.75 | 0.85 | 1 |
| C310 | 0 | 0.27 | 0.45 | 0.73 | 0.18 | 0.49 | 0.69 | 1 | 0 | 0.27 | 0.45 | 0.73 | 0.18 | 0.49 | 0.69 | 1 |
| C311 | 0 | 0.13 | 0.21 | 0.33 | 0.44 | 0.65 | 0.79 | 1 | 0.33 | 0.52 | 0.65 | 0.83 | 0.44 | 0.65 | 0.79 | 1 |
| C41 | 1 | 0.96 | 0.93 | 0.89 | 0.41 | 0.26 | 0.15 | 0 | 0.53 | 0.40 | 0.31 | 0.18 | 0.53 | 0.40 | 0.31 | 0.18 |
| C42 | 1 | 0.74 | 0.56 | 0.30 | 0.80 | 0.50 | 0.30 | 0 | 1 | 0.74 | 0.56 | 0.30 | 1 | 0.74 | 0.56 | 0.30 |
| C43 | 0 | 0.13 | 0.22 | 0.36 | 0.29 | 0.47 | 0.60 | 0.79 | 0.43 | 0.64 | 0.79 | 1 | 0.43 | 0.64 | 0.79 | 1 |
| C44 | 0 | 0.19 | 0.31 | 0.50 | 0.33 | 0.58 | 0.75 | 1 | 0.33 | 0.58 | 0.75 | 1 | 0.33 | 0.58 | 0.75 | 1 |
| C45 | 0 | 0.15 | 0.25 | 0.40 | 0.13 | 0.31 | 0.43 | 0.60 | 0.40 | 0.63 | 0.78 | 1 | 0.40 | 0.63 | 0.78 | 1 |
