# Supplementary material for: A novel fuzzy framework for technology selection of sustainable wastewater treatment plants based on TODIM methodology in developing urban areas
Source: Sci Rep. 2022 May 25;12:8800. doi: 10.1038/s41598-022-12643-1 (PMC9132933; doi:10.1038/s41598-022-12643-1)
Supplement: Supplementary file 6 — Supplementary Table 6. [file 41598_2022_12643_MOESM6_ESM.docx]

**Supplementary Table 6.** Normalized weights matrix of evaluated objective data calculated through Eq. (19) and Eq. (20).

| α-cuts | | | | | | | | | | | |
| --- | --- | --- | --- | --- | --- | --- | --- | --- | --- | --- | --- |
|  | *0* | *0.1* | *0.2* | ***0.3*** | *0.4* | *0.5* | *0.6* | *0.7* | *0.8* | *0.9* | *1.0* |
| Criteria |  |  |  |  |  |  |  |  |  |  |  |
| C11 | 0.058 | 0.117 | 0.011 | 0.014 | 0.052 | 0.126 | 0.0125 | 0.0144 | 0.0505 | 0.136 | 0.0132 |
| C12 | 0.248 | 0.080 | 0.011 | 0.138 | 0.223 | 0.084 | 0.0117 | 0.151 | 0.2389 | 0.090 | 0.0120 |
| C13 | 0 | 0.084 | 0.000236 | 0.000335 | 0 | 0.082 | 0.00028 | 0.0004 | 0 | 0.079 | 0.000426 |
| C21 | 0.025 | 0.015 | 0.044 | 0.007 | 0.030 | 0.021 | 0.056 | 0.010 | 0.034 | 0.024 | 0.062 |
| C22 | 0.010 | 0.058 | 0.048 | 0.115 | 0.009 | 0.0608 | 0.0512 | 0.121 | 0.009 | 0.063 | 0.054 |
| C23 | 0.053 | 0.081 | 0.012 | 0.043 | 0.048 | 0.086 | 0.014 | 0.045 | 0.047 | 0.089 | 0.015 |
| C24 | 0.016 | 0 | 0.012 | 0.037 | 0.013 | 0 | 0.012 | 0.035 | 0.012 | 0 | 0.012 |
| C25 | 0.209 | 0.082 | 0.012 | 0.141 | 0.182 | 0.086 | 0.012 | 0.152 | 0.187 | 0.091 | 0.012 |
| C31 | 0.003 | 0.006 | 0.008 | 0.011 | 0.012 | 0.013 | 0.018 | 0.015 | 0.017 | 0.016 | 0.024 |
| C32 | 0 | 0.015 | 0.014 | 0.023 | 0.006 | 0.019 | 0.021 | 0.025 | 0.001 | 0.021 | 0.026 |
| C33 | 0.012 | 0.017 | 0.006 | 0.017 | 0.018 | 0.021 | 0.014 | 0.021 | 0.022 | 0.023 | 0.019 |
| C34 | 0.029 | 0.041 | 0.002 | 0.019 | 0.029 | 0.041 | 0.006 | 0.021 | 0.029 | 0.040 | 0.009 |
| C35 | 0.06 | 0.017 | 0.008 | 0.002 | 0.055 | 0.020 | 0.013 | 0.004 | 0.051 | 0.021 | 0.017 |
| C36 | 0 | 0.024 | 0.084 | 0.025 | 0.003 | 0.026 | 0.084 | 0.026 | 0.005 | 0.026 | 0.081 |
| C37 | 0 | 0.017 | 0.006 | 0.019 | 0.007 | 0.021 | 0.014 | 0.022 | 0.011 | 0.022 | 0.019 |
| C38 | 0 | 0.074 | 0.144 | 0.072 | 0.00036 | 0.069 | 0.135 | 0.066 | 0.00072 | 0.065 | 0.128 |
| C39 | 0 | 0.074 | 0.143 | 0.071 | 0.0004 | 0.069 | 0.134 | 0.066 | 0.00072 | 0.064 | 0.128 |
| C310 | 0 | 0.0157 | 0.006 | 0.018 | 0.008 | 0.020 | 0.015 | 0.021 | 0.012 | 0.022 | 0.020 |
| C311 | 0 | 0.046 | 0.061 | 0.045 | 0.003 | 0.045 | 0.061 | 0.043 | 0.004 | 0.042 | 0.061 |
| C41 | 0.25 | 0.006 | 0.033 | 0.016 | 0.22 | 0.006 | 0.032 | 0.016 | 0.22 | 0.006 | 0.031 |
| C42 | 0.03 | 0.005 | 0.045 | 0.024 | 0.036 | 0.009 | 0.053 | 0.027 | 0.038 | 0.011 | 0.057 |
| C43 | 0 | 0.025 | 0.089 | 0.043 | 0.003 | 0.026 | 0.084 | 0.041 | 0.005 | 0.026 | 0.080 |
| C44 | 0 | 0.031 | 0.064 | 0.032 | 0.005 | 0.0322 | 0.064 | 0.032 | 0.007 | 0.032 | 0.063 |
| C45 | 0 | 0.009 | 0.082 | 0.04 | 0.003 | 0.012 | 0.077 | 0.038 | 0.006 | 0.013 | 0.074 |
