# Supplementary material for: A novel fuzzy framework for technology selection of sustainable wastewater treatment plants based on TODIM methodology in developing urban areas
Source: Sci Rep. 2022 May 25;12:8800. doi: 10.1038/s41598-022-12643-1 (PMC9132933; doi:10.1038/s41598-022-12643-1)
Supplement: Supplementary file 7 — Supplementary Table 7. [file 41598_2022_12643_MOESM7_ESM.docx]

**Supplementary Table 7**

Requirements for discharges from urban wastewater treatment plants subject to Articles 8 of the Urban Wastewater Treatment Directive of Turkey.

| Parameter | Concentration, mg/L | Minimum Percentage of Reduction |
| --- | --- | --- |
| 5-day Biochemical Oxygen Requirement, BOD_5_, mg/L | 25 | 70-90 |
| Chemical Oxygen Demand, COD, mg/L | 125 | 75 |
| Total Suspended Solids, TSS, mg/L | 35  35 (more than 10,000 p.e.)  60 (2,000-10,000 p.e.) | 90  90  70 |
|  |  |  |

**Supplementary Table 8**

Requirements for discharges from urban waste water treatment plants to sensitive areas which are subject to eutrophication as identified in Article 4 of the Urban Wastewater Treatment Directive of Turkey. One or both parameters may be applied depending on the local situation. The values for concentration or for the percentage of reduction shall apply.

| Parameter | Concentration, mg/L | Minimum Percentage of Reduction |
| --- | --- | --- |
| Total nitrogen, mg/L | 15 mg/1 N (10 000 - 100 000 p.e.)  10 mg/1 N (more than 100 000 p.e) | 70-80 |
| Total phosphorus, mg/L | 2 mg/1 P (10 000 - 100 000 p.e.)  1 mg/1 P (more than 100 000 p.e.) | 80 |
